# Dietary fiber intake impacts gut bacterial and viral populations in a hypertensive mouse model

**DOI:** 10.1080/19490976.2024.2407047

**Published:** 2024-09-28

**Authors:** Laura Avellaneda-Franco, Liang Xie, Michael Nakai, Jeremy J. Barr, Francine Z. Marques

**Affiliations:** a School of Biological Sciences, Monash University, Melbourne, Australia; b Precision Medicine Translational Research Programme, Department of Obstetrics & Gynaecology, Yong Loo Lin School of Medicine, National University of Singapore, Singapore, Singapore; c Heart Failure Research Group, Baker Heart and Diabetes Institute, Melbourne, Australia; d Victorian Heart Institute, Monash University, Melbourne, Australia

**Keywords:** Microbiome, hypertension, angiotensin II model, gut virome, prophage, fiber intake, CAZymes

## Abstract

The gut microbiome is an emerging factor in preventing hypertension, yet the influence of gut bacteriophages, viruses infecting bacteria, on this condition remains unclear. Bacteriophage-bacteria interactions, which impact the gut microbiome, are influenced differentially by temperate and virulent bacteriophages. However, the standard technique for studying viral populations, viral-like particles (VLPs)-metagenomes, often overlook prophages, the intracellular stage of temperate bacteriophages, creating a knowledge gap. To address this, we investigated alterations in extracellular and intracellular bacteriophages, alongside bacterial populations, in the angiotensin II-hypertension model. We sequenced VLPs and bulk DNA from cecal-colonic samples collected from male C57BL/6J mice implanted with minipumps containing saline or angiotensin II. We assembled 106 bacterial and 816 viral genomes and found that gut viral and bacterial populations remained stable between hypertensive and normotensive mice. A higher number of temperate viruses were observed across all treatments. Although temperate viruses outnumbered virulent viruses, sequencing of both VLPs and bulk revealed that virions from virulent viruses were more abundant in the murine gut. We then evaluated the impact of low- and high-fiber intake on gut microbiome composition in the angiotensin II model. Fiber intake significantly influenced the gut microbiome composition and hypertension development. Mice receiving high-fiber had lower blood pressure, a higher bacterial-encoded carbohydrate-associated enzyme, and a higher total relative abundance of temperate viruses than those receiving low-fiber. Our findings suggest that phages are not associated with hypertension development in the angiotensin II model. However, they support a complex diet-bacteria/phage interaction that may be involved in blood pressure regulation.

## Introduction

The human gut microbiome, which comprises bacteria, viruses, eukaryote microbial cells, and their metabolic by-products, has emerged as a potential contributor to blood pressure (BP) regulation.^1–5^ High BP, also known as hypertension, is a leading global cause of morbidity and mortality, contributing to 10.8 million deaths in 2019.^4,6^ Lifestyle changes, particularly dietary modifications, are considered first-line therapy for hypertension.^7^ Among these dietary changes, increasing fiber intake is recommended due to its ability to lower BP.^8^ Evidence from rodent hypertensive models suggests that high fiber diets confer a protective phenotype, resulting in reduced arterial BP compared to low-fiber diet, alongside alterations in gut bacterial populations.^9^ An important yet understudied component of the microbiome is viruses. Although differences in gut viral populations have been observed between hypertensive patients and healthy individuals,^10^ the effect of fiber intake on viral populations in hypertension remains unknown.

Gut viral populations are predominantly composed of bacteriophages,^11–14^ which are viruses that infect bacteria and are collectively referred to as phages. Phages may serve as therapeutic tools to tune the gut microbiome due to their potential to modulate the gut bacterial community structure and functionality.^15–17^ The effect of phages on the bacterial community depends on, among others, phages’ lifestyles.^18,19^ Based on their lifestyle, phages are classified into temperate and virulent phages.^20^ Virulent phages can impact gut bacterial populations and their metabolic network through the lytic cycle,^17^ wherein they lyze the bacterial cell to release virions.^20^ Alternatively, temperate viruses, capable of undergoing both lysogenic and lytic cycles,^20^ can either lyze the bacteria or alter bacterial metabolism.^11,12,21^ During lysogenic cycles, prophages, the intracellular stage of temperate phages, replicate alongside their bacterial hosts until a trigger prompts the induction of the prophage, initiating reentry into the lytic cycle.^20^ Dietary compounds such as stevia and bee propolis, as well as dietary-derived gut metabolites such as short-chain fatty acids (SCFAs), can trigger the induction of gut prophages.^22,23^ The induction of prophages in the gut is hypothesized to significantly influence the assembly and homeostasis of the gut microbiome.^23–26^

To leverage phages as a therapeutic tool in hypertension, we need first to characterize the virome in experimental hypertension. Characterizing gut phages, including prophages, presents a challenge because, unlike bacteria, phages lack phylogenetic markers, such as *rpoB* and 16S rRNA, in their genomes.^14,27^ Hence, metagenomic approaches like viral-like particles (VLPs) metagenomes are required to study viral populations. Although VLP metagenomes are the standard approach to studying the gut virome, they fail to capture prophages.^18,28^ As a result, only phages undergoing the lytic cycle are represented in the assembled viral contigs, neglecting resident prophages. In contrast, whole microbial (bulk) metagenomes can capture both states of temperate phages (i.e., prophages and induced virions) and have a higher detection performance of viral populations if searched with a well-furnished database.^11^ However, the absence of a publicly available murine gut viral database means that the reconstruction of prophage genomes from bulk metagenomes relies on phage prediction software. These technical limitations have hindered comprehensive studies, with only one study exploring fluctuations in viral populations in human hypertension from metagenomes^10^ and none exploring these fluctuations in experimental rodent models of hypertension.

Here, we aimed to evaluate the impact of angiotensin II (Ang II), the most-used mouse model of hypertension,^29^ and dietary fiber interventions on both temperate and virulent viral populations, alongside bacteria, in wild-type C57BL/6J mice. To achieve this, we first reconstructed bacterial and both virulent and temperate viral genomes using a dual-source metagenomics strategy that combines the strengths of VLP and bulk metagenomes. We hypothesized that bulk metagenomes would capture bacterial and viral populations, including temperate and virulent phages, while VLP genomes would be more sensitive in capturing viruses in the lytic cycle. By employing this dual-source metagenomics approach in the Ang II model, we constructed a local collection of gut murine bacterial and viral genomes. This collection enabled us to evaluate the effect of diet on the gut-reconstructed microbial populations in experimental hypertension while providing a robust methodology to capture the various lifestyles of gut viruses. This methodology can be applied to other experimental murine models and human diseases.

## Methods

### Animal experiments

The Ang II mouse model, the most used model of experimental hypertension,^29^ on the C57BL/6J background was used. Six-week-old male wild-type C57BL/6J mice received a subcutaneous minipump (Alzet model 2004) placed during surgery under anesthesia with isoflurane containing either vehicle (0.9% saline, Sham-control) or Ang II (0.5 mg/kg body weight/day; Auspep) for 4 weeks (total 8 mice, *n* = 4/group). All the mice were fed a control diet (AIN93G). In addition, samples from a previous experiment^30^ of 6-week-old male wild-type C57BL/6J randomized to a diet lacking resistant starches (low resistant starch/fiber, LF, SF09–028, Specialty Feeds) or a diet rich in resistant starches (high resistant starch/fiber, where all carbohydrates were replaced with resistant starches, HF, SF11–025, Specialty Feeds), both formulated on the background of the AIN93G diet, after minipump implantation containing Ang II (total 8 mice, *n* = 4/group) were used to study the role of diet in the gut microbiome. All samples were collected from mice under animal ethics approval 17,465 from Monash University.

### Blood pressure measurements

Systolic BP was measured using tail-cuff in a CODA noninvasive BP system (Kent Scientific Corporation). Prior to baseline and weekly measurements during the 4-week mini pump protocol, mice were acclimatized for 3 days. During the measurements, five acclimatization cycles and recorded 10 measurement cycles were performed, where at least 4 acceptable measurements were used to calculate the average BP per animal per week.

### Bulk and VLPs dsDNA extraction and sequencing

Mice were euthanized with CO_2_, and the cecal and colonic content (representative of the whole large intestine) were collected and mixed. To characterize bacterial and double-stranded DNA (dsDNA) viral populations across the samples, we performed whole-genome (bulk) and VLP shotgun sequencing samples from the whole large intestine. We processed 0.2 g of fresh sample to extract nucleic acids from VLPs on the same day at the time of euthanasia, while the remaining sample was stored at −80°C. Total genomic DNA was extracted using the QIAamp PowerFecal Pro DNA isolation kit (Qiagen) following the suppliers’ protocol.

VLPs-enrichment and the extraction of nucleic acids from VLPs were performed following a hybrid protocol adapted from previously described methods.^14,31,32^ Briefly, we resuspended 0.2 g of sample in 1.2 mL of sterile SM buffer, vortexed it for 5 min, and centrifuged twice at 7,000 g for 10 minutes.^14,31,32^ Supernatant was collected and filtered through a 0.45 μm pore size membrane filter. Next, the filtrate was treated with 1 mL of chloroform for 5 min and centrifuged at 12,500 g for 30 minutes at 4 ∘C. To digest free DNA, we treated the supernatant with 55.5 μ L of 50 mm MgCl_2_ and 10 mm CaCl_2_ solution, 5.5 μ L of DNAse I, and 55 μ L of RNAse A for 1 hour at 37 ∘C. The digestion was stopped by incubating the samples for 10 min at 70 ∘C.

To extract nucleic acid from the VLPs, we added 2.5 μ L of proteinase K and 25 μ L of 10% SDS solution to the samples and incubated them for 20 minutes at 56 ∘C. After this, we added 125 μ L of lysis buffer (4.5 M guanidium isothiocyonate, 44 mm citrate pH 7.0, 0.88% sarkosyl, 0.72% 2-mercaptoethanol) and incubated the samples for 10 minutes at 65 ∘C. To purify the nucleic acids, the samples were treated twice with an equal volume (700 μL) of phenol:chloroform:isoamyl alcohol 25:24:1, followed by incubation on ice for 5 minutes, and centrifugation at 8,000 g for 5 minutes at 4 ∘C. To precipitate the nucleic acids, 60 μ L of 3 M sodium acetate and 1.2 mL of ethanol were added to the aqueous phase followed by centrifugation at 8,000 g for 5 minutes at 4 ∘C. Finally, the pellet was washed with 1 mL 70% ethanol, which was centrifuged at 8,000 g for 5 minutes at 4 ∘C. Evaporation was allowed, and the pellets were resuspended in 50 μ L of ultrapure water.

### Sequencing, quality control, and contigs assembly

The extracted bulk and VLPs dsDNA were sequenced using an Illumina NovaSeq 6000 sequencer (2×150 bp). The obtained reads were filtered by quality using Trimmomatic v0.38 (HEADCROP:10 SLIDINGWINDOW:4:20 MINLEN:60).^33^ In addition, the trimmed reads were mapped against the human (GRCh38) and the mouse (GRCm39) genomes using bowtie2 v2.3.5.^34^ Reads that mapped against any of these genomes were filtered out. Finally, we assembled the quality-reads into contigs per library using metaSPAdes v3.13.1.^35^

### Identifying bacterial species-level representative genomes (SRGs)

Assembled contigs of each bulk library were binned in metagenome-assembled genomes (MAGs) using MetaBAT v2.15.5.^36^ Quality-reads of each library were then mapped against the obtained MAGs using bowtie2 v2.3.5.^34^ The unmapped reads were used for a second round of assembly, where the obtained contigs were binned into MAGs as before. MAGs from both assembly rounds across samples were dereplicated into a set of species-level representative genomes (SRGs) using dRep v3.0.0 (-comp 50 -con 10 -nc 0.25).^37^ Each SRG was taxonomically assigned according to the Genome Taxonomy Database (GTDB) (release 207_v2)^38^ using GTDB-tk v2.1.0.^39^ Finally, bacterial genes of each SRG were predicted and annotated using Distilled and Refined Annotation of Metabolism (DRAM).^40^

### Identifying viral species-level representative genomes (SRGs) from bulk and VLPs metagenomes

Raw potential viral genomes were obtained from three sources: VirSorter v1.0.5 (–db 2 –virome – diamond)^41^ predictions from MAGs, VLPs contigs, and bulk unbinned contigs. The completeness of potential viral genomes was evaluated using CheckV v0.7.0.^42,42^ Genomes with at least 50% of completeness were concatenated and dereplicated into SRGs at a 99% identity over a local alignment of 100% of the shortest sequence using CD-HIT v4.8.1 (-c 0.99 -G 0 -aS 1).^43^ The obtained representative viral sequences were then validated and annotated using VirSorter/DRAM-v^40,41^ and VIBRANT^44^ phage predictors. Sequences predicted neither by VirSorter nor VIBRANT were filtered out. Moreover, contigs without at least one of these structural proteins: terminase, capsid, tail, N-acetylmuramoyl-L-alanine amidase, baseplate, prohead, coat, or virion were excluded. In addition, contigs codifying for only one structural protein and encoding more than 100 open read frames (ORFs) were also discarded. Viruses that presented genes associated with lysogenic cycles (integrase, transposase, recombinase, and Cro/CI domains) or homology with bacterial SRGs coupled with the presence of flanked bacterial regions were cataloged as predicted temperate phages, and the rest of them were cataloged as predicted virulent viruses. We are aware that this classification can lead to misclassification of fragmented viral genomes or phages with novel mechanisms to perform lysogenic cycles. However, this is a limitation of our current knowledge about gut phages.

### Determining the impact of sequencing-depth in the recovery of bacterial and viral single-representative genomes (SRGs)

To determine the sequencing depth to completely survey the whole microbiome and virome in a cecal/colon mixed sample, we performed rarefactions curves of (i) bacterial and (ii) viral SRGs using both bulk and VLP metagenomes from a sample of a five-week healthy C57BL/6J mouse. Briefly, we extracted and sequenced dsDNA from bulk and VLPs from a mixed caecal/colon sample of one healthy C57BL/6J mouse following the above-mentioned methods. We obtained 28,388,204 and 19,430,023 paired-end (PE) reads from the bulk and VLP libraries, respectively. Then, we subsampled in triplicates: 1, 5, 10, 15, 20, and 25 million PE reads from the bulk, and 1, 5, 10, and 15 million PE reads from the VLP metagenomes using seqtk v.1.3.^45^ Next, we assembled and binned each bulk subsampled-library into bacterial SRGs, and we assembled and identified viral SRGs from VLPs and bulk subsampled libraries. Finally, the number of viral and bacterial SRGs across the sampling size was plotted to check the number of reads necessary to reach a plateau.

### Constructing a collection of murine gut viral and bacterial genomes

To reconstruct gut viral and bacterial genomes from mice, we identified bacterial and viral SRGs according to the above-explained methods using the samples across the two experiments (Sham-control vs Ang II, low vs high fiber diets) in this study. Therefore, we used 16 bulk and 8 VLPs metagenomes from 16 mice to recover the murine gut viral and bacterial genomes.

### Viral and bacterial population profiling

To calculate the raw abundances of the viral and bacterial populations in each sample, reads from the bulk and VLPs of each sample were mapped against the bacterial and viral SRGs collection using bowtie2 v2.3.5.^34^ A summary of the mean depth and coverage of each SRG was calculated using “samtools coverage” from Samtools v.1.15.1.^46^ To filter out SRGs without enough coverage across their length, we established the following thresholds to viral and bacterial SRGs. Viral SRGs were considered detected when either ≥70% or at least 5,000 bp of the SRGs was covered.^11^ For bacterial populations, we considered detected when either ≥70% or at least 100,000 bp of the SRG was covered. Viral and bacterial SRGs present in more than 2 samples were kept and raw abundance was obtained from the rounded mean depth per sample. These raw abundances’ tables were converted into phyloseq objects for further analysis.^47^

### Functional bacterial profiling

To calculate the raw abundance of each bacterial gene, reads from bulk metagenomes of each sample were mapped against DRAM’s predicted ORFs using bowtie2 v2.3.5.^34^ A summary of the mean depth and coverage of each SRG was calculated using Samtools v.1.15.1.^46^ Only genes with a length coverage higher than 70% were considered as detected in each sample. The mean depth was used as the raw abundance of each gene. Finally, the abundances of genes belonging to the same gene_ID given by DRAM were summed up together as the raw abundance of each gene. A phyloseq object was constructed for further analysis. KEGG IDs: K00128, K00149, K00138, K14085, K00129, K24012, K01905, K22224, K00925, K19670, K02576, K02577, K02578, K18118, K01026, K01067, K01895, K01913, K00467, K00156, K01512, K10150, K01738, K13034, and K17069 were associated with acetate production. K01034, K01035, K19709, K23756, K01896, K01913, K00929, K00634 with butyrate, and K00925, K00932, K19697, K01895, K01908, K20454 with propionate.

### Statistical analysis

All the statistical analyses were conducted using R v.4.2.2^48^ in RStudio.^49^ In all the cases, Shapiro–Wilk’s test and Levene’s test were performed to check the normal distribution of the residuals and the homogeneity of the variances of each dataset. For two-group comparisons, a two-tail *t*-tests were used for normally distributed variables, while nonparametric Wilcoxon-Mann-Whitney test was used in the remaining cases. Kruskal–Wallis test with Dunn’s post-hoc test was used for multiple comparisons were normality and homoscedasticity were not fulfilled, otherwise two-way analysis of variance (ANOVA) with Tukey’s post-hoc test. False discovery rate (FDR) was used to correct p-values from multiple comparisons, with significance defined as adjusted *p* < .05. Analyses of alpha and beta diversity were performed using phyloseq v.1.42.0 and vegan v.2.6-4 packages. PERMANOVA analyses using Bray-Curtis distances, and 9,999 permutations were used to test for differences in the viral/bacteria composition across treatments using vegan v.2.6-4 package. The correlation among viral and bacterial communities from Bray-Curtis based principal component using Procrustes randomization test with 9,999 permutations. Differential abundant features, such as viral and bacterial SRGs, and bacterial encoded genes, between treatments were determined using dar 0.99.0.^50^ Using dar, we obtained a set of differentially abundant features from the consensus of DeSeq, MaAsLin, and metagenomeSeq outputs. Using the log2 fold change (log2FC) from DeSeq, we filtered out viral and bacterial SRGs, as well as genes, that have a |log2FC| <2. Thus, differentially abundant genes or populations here reported are the consensus of these three tests and have a | log2FC |>2 with FDR-adjusted *p* < .05.

## Results

### A dual-source metagenomics strategy to capture gut bacterial and viral populations

**Figure 1.**
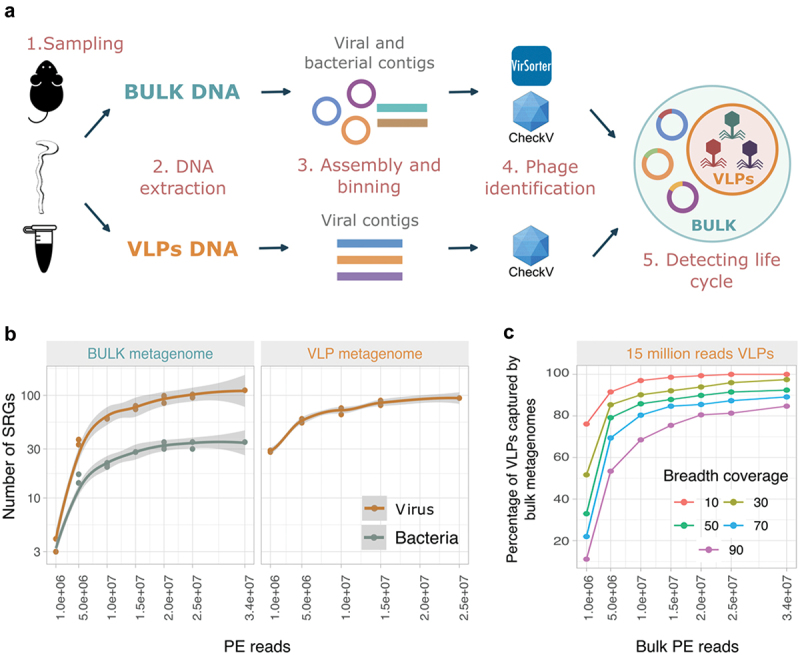
A dual-source metagenomics approach to efficiently identify gut viral populations was validated. a, the approach combines whole microbial (bulk) and viral-like particles (VLPs) metagenomes and involves five main steps. First, a mixed sample of the contents of the large intestine (cecum and colon) was collected from each mouse at time of death. Second, the sample was used for DNA extractions from both bulk and VLPs. Third, high-quality reads were assembled into contigs. In addition, bacterial genomes were obtained through binning of the bulk contigs. Fourth, viruses were identified using VirSorter, VIBRANT, and CheckV. Only viral contigs with a completeness higher than 50% and structural genes were cataloged as viral species-level representative genomes (SRGs). Fifth, temperate and virulent viruses were predicted based on protein content. b, the impact of the sequencing depth on the recovery of viral and bacterial SRGs was evaluated. The gray areas denote the 95% confidence interval. c, using a VLP metagenome with 15 million PE reads, we evaluated the effect of the bulk depth sequencing and breadth coverage on the percentage of viral SRGs from the VLP metagenomes that are captured in the bulk metagenome. A viral SRG was cataloged to be present in the sample if it was covered by at least a set breadth coverage threshold.

To evaluate the impact of sequencing depth on the recovery of viral and bacterial populations when using dual-source metagenomes, we extracted and sequenced DNA from both VLP and whole microbial (bulk) fractions. These extractions were performed using a mixed sample of cecal and colonic contents from a five-week-old mouse (Figure 1a). The obtained reads from each metagenome were randomly subsampled in subsets of different sequencing depth sizes, and for each subset, we identified viral and bacterial species-level representative genomes (SRGs). We observed that the number of viral and bacterial SRGs reached a plateau after 15 and 20 million paired end (PE) reads from VLPs and bulk metagenomes, respectively (Figure 1b). Therefore, we set 15 and 20 million PE reads as minimum sequencing depth to comprehensively assess the murine gut bacterial and viral populations when using the dual-source metagenomic approach.

Although bulk metagenomes also reached a plateau in terms of detected viral SRGs, viral SRGs recruited only a maximum of 10.2% of the bulk reads. This low level of viral reads within the bulk metagenome may lead to a reduction in the breadth coverage of each viral genome. Given we used the breadth coverage to define the presence of a given SRG, we did investigate the impact of the breadth coverage threshold and the sequencing depth on the sensitivity of bulk metagenomes in capturing viral SRGs reconstructed from VLPs metagenomes. Our goal was to set the minimum breadth coverage and sequencing depth of the bulk metagenome that could accurately represent the majority of detected VLPs in a 15 million reads viral metagenome. To achieve this, we assessed the viral SRGs recovered by one subset of each of the previously tested sequencing depth sizes of the bulk metagenomes using five different length coverage thresholds. We then compared the quantity of bulk recovered VLPs with the number of viral SRGs reconstructed from the 15 million PE reads VLP metagenome. The breadth coverage threshold influenced the percentage of viral-SRGs from VLP metagenomes covered by the bulk metagenomes when using less than 20 million PE reads (Figure 1c). However, with over 20 million PE reads, over 80% of the viral SRGs identified in VLP metagenomes were found in the bulk sample, regardless of the breadth coverage threshold. Based on these findings, we set the breadth coverage threshold of 70% and used 20 million PE reads from the bulk for further experiments, as this combination captured 85% of the viral-SRGs from VLPs in the bulk. Thus, the dual-source metagenomic approach allowed us to fully reconstruct a collection of gut viral SRGs that successfully surveyed over 80% of viral-SRGs reconstructed from the VLPs in bulk metagenomes.

### A collection of gut murine microbial populations comprising 106 bacterial and 816 viral SRGs was constructed

We constructed a collection of bacterial and viral SRGs from the murine gut using the dual-source metagenomic approach. For this, we sequenced 16 bulk and 8 VLP metagenomes from mixed samples of cecal and colonic content of 16 mice. After removing mouse and human DNA reads, we obtained 26,131,652 ± 9,972,309 and 29,388,180 ± 3,085,480 PE reads from bulk and VLP metagenomes, respectively. The number of final reads per sample exceeded the plateau obtained in the rarefaction curves (i.e. 15 and 20 million PE reads from VLPs and bulk metagenomes, respectively), enabling us to comprehensively reconstruct the bacterial and viral populations. Using these reads, we reconstructed 106 bacterial and 816 viral SRGs with a genome completeness greater than 50% and a genome contamination less than 10%. The bacterial SRGs were classified according to their respective phyla, with the majority (75) belonging to the Bacillota phylum, followed by (21) in the Bacteroidota phylum, and smaller numbers in the Actinomycetota (3), Desulfobacteriota (3), Pseudomonadota (2), Deferribacterota (1), and Verrucomicrobiota (1) phyla. Furthermore, 671 (82.2%) of the viral SRGs were classified into known taxonomical phage families, and 480 of these were predicted as temperate SRGs based on the presence of integrase, transposase, recombinase, and Cro/CI like domains. The presence of these genes was also found in 100 of the not taxonomically assigned viral SRGs. The remaining 236 viral SRGs lack these genes and are hereafter referred to as virulent SRGs for readability. However, we acknowledge that this classification strategy can lead to misclassification of fragmented viral genomes, as temperate phages that do not integrate into the host, novel phages, or phage-encoded proteins with these domains may have different functions. Finally, the total viral-SRGs were grouped into 178 viral clusters (VCs) and 107 singletons.

### The gut virome and bacteriome did not change between normotensive and Ang II-induced hypertensive mice

**Figure 2.**
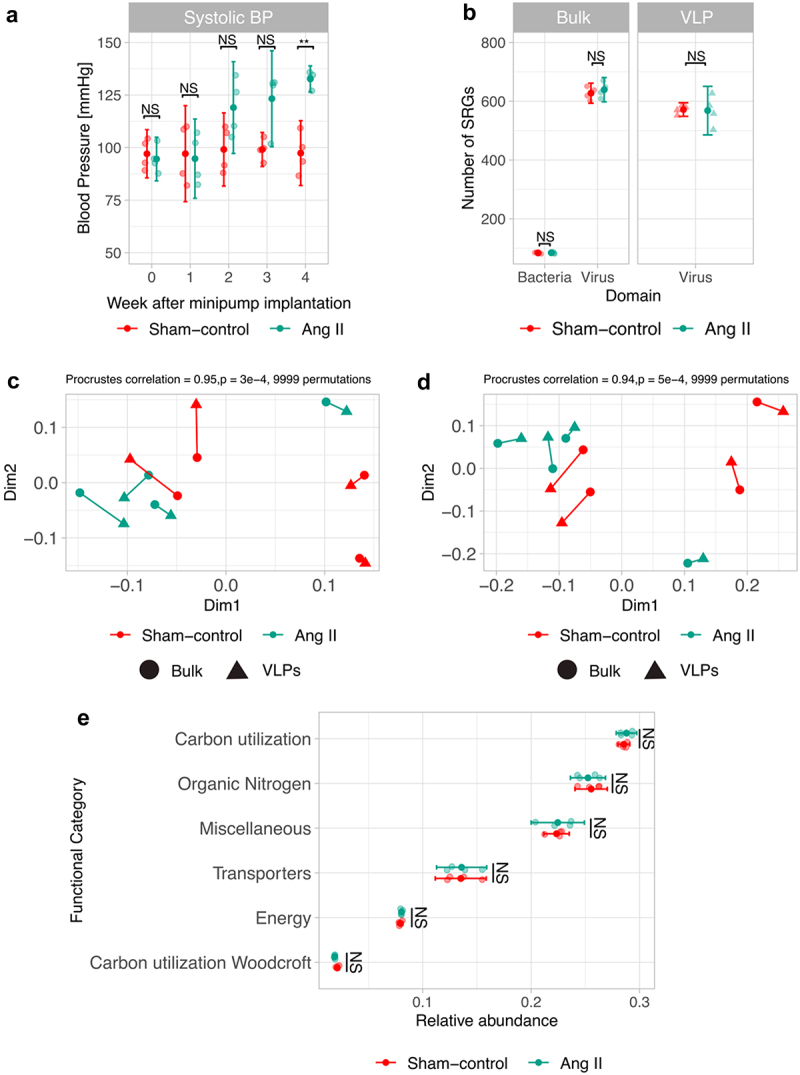
Gut bacterial and viral populations, as well as bacterial genes diversity, did not change between normotensive and Ang II-driven hypertensive mice. After 4-weeks of implantation of a minipump either with saline (Sham-control) or Ang II, a, systolic blood pressure (BP) of Ang II challenged mice was higher compared to Sham-control mice. The development of hypertension using the Ang II model did not impact either b, the richness of gut bacterial and viral populations, nor the bacterial and viral composition. However, a strong correlation was found between the viral and bacterial community composition in both VLPs and bulk metagenomes. Procrustes rotation of the viral community PCoA coordinates from c, VLPs or d, bulk metagenomes versus bacterial community PCoA coordinates; Bray Curtis metric was used in all cases. e, No significant differences across DRAM-v gene functional categories were observed in the gene repertoire of bacterial populations on Ang II-challenged mice compared to Sham-control mice. N = 4 mice/group. *p<.05; **p<.01; ***p<.001; NS p ≥ 0.05. Two-way ANOVA with Tukey post hoc test (a), two-pair group t-test (b), and pairwise t-test with FDR corrections for multiple testing (e). The dark points represent the mean, the whiskers represent the 95% CI, and the translucid dots represent the measure of each mouse (a, b, e). Each dot represents the measure of each mouse (c-d).

We employed the dual-source metagenome approach to evaluate potential changes in the gut microbiome composition following the induction of hypertension in male C57BL/6J mice using the Ang II model. Briefly, eight littermate mice were randomized to receive either Ang II or saline (Sham-control) via a minipump and their BP was monitored weekly for 4 weeks. At the end of the fourth week, we extracted and sequenced bulk and VLP DNA from the large intestine contents of each mouse. Compared to Sham-control mice, mice receiving Ang II exhibited a significantly higher systolic BP (+35 mmHg, *p* = .0015) at week 4 (Figure 2a), demonstrating the induction of hypertension.

Based on the gut bacterial (Supplementary Figure 1) and viral composition (Supplementary Figure 2), we found no significant differences in either α-diversity observed SRGs of either bacterial (*p* = .818), bulk viral (*p* = .5408), or VLP viral populations (*p* = .9322) (Figure 2b) nor in ACE, Chao1, Simpson, or Shannon index (Supplementary Figure 3). Similarly, we did not find differences in β-diversity measures of either bacterial (*p* = .1454), bulk viral (*p* = .6552), or VLP viral populations (*p* = .5082) (Figure 2c,d) between Sham-control and Ang II mice. In addition, we did not observe differential abundance in individual bacterial-encoded genes nor significant differences across bacterial functional categories based on DRAM^40^ (Figure 2e, see summary statistics in Supplementary Table 1). These findings suggest that hypertension induced by Ang II did not significantly impact the gut viral and bacterial populations in male C57BL/6J mice.

We did observe a strong correlation between the composition of the gut bacterial community and the viral community surveyed from both bulk (*r* = 0.94, *p* = 5e-4) and VLP (*r* = 0.95, *p* = 3e-4) metagenomes (Figure 2c,d) using a Procrustes rotation of the bacterial community PCoA coordinates versus viral community PCoA coordinates. This correlation suggested that viral populations may shape or mirror the structure and function of the bacteriome. In addition, we found a small correlation between the abundances of viral SRGs from the bulk and the VLPs metagenomes (*r* = 0.19, *p* < 2.2e-16), indicating that bulk and VLPs screen different components of the human virome, highlighting the importance of studying both metagenomes to completely understand the role of viruses in the gut.

### Temperate viruses outnumbered virulent viruses; however, virulent virus virions were more abundant

**Figure 3.**
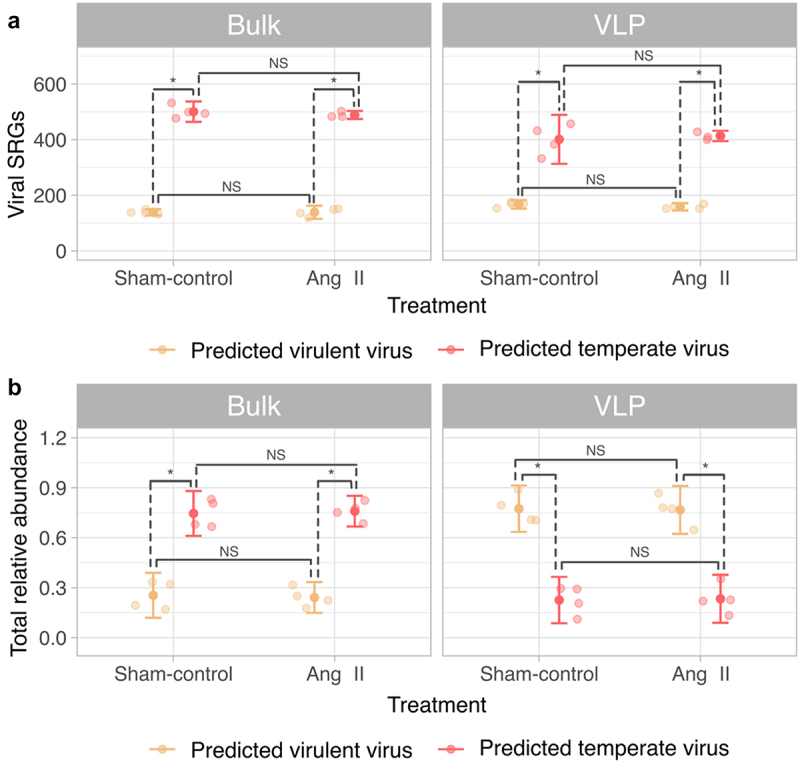
Number and total relative abundance of viruses based on their lifestyle. a, predicted temperate viruses outnumber virulent viruses regardless of the tested metagenome or treatment. b, the larger number of temperate viruses is positively correlated with the higher total relative abundance of these viruses in the bulk (r(8) = 0.82, p = 9.2e-5), but it is negatively correlated with the abundance of these phages in the VLP metagenome (r(8)=-0.75, p = 8.4e-4). Thus, virulent phages are more abundant in the VLP metagenomes. N = 4 mice/group. *p<.05; **p<.01; ***p<.001; NS p ≥ 0.05. Pairwise Wilcoxon test with FDR corrections for multiple comparisons. The dark points represent the mean, the whiskers represent the 95% CI, and the translucid dots represent the measure of each mouse.

To investigate the differences in gut temperate and virulent viral populations between the bulk and VLP metagenomes, we calculated the number of temperate and virulent viral SRGs alongside their total relative abundance in both metagenomes. We observed that temperate viruses outnumbered virulent viruses in both bulk (*p* = .029) and VLP (*p* = .029) metagenomes (Figure 3a), but their total relative abundance had an opposite pattern between metagenomes. While the number of temperate viruses positively correlated with their total relative abundance in bulk metagenomes (r(8) = 0.82, *p* = 9.2e-5), it negatively correlated with their total relative abundance in the VLP metagenomes (r(8)=-0.75, *p* = 8.4e-4). Thus, in bulk metagenomes, where temperate viruses are captured from lytic and lysogenic cycles, the total relative abundance of temperate viruses was significantly higher than the total relative abundance of virulent viruses (Figure 3b). This higher abundance of temperate viruses in bulk metagenomes was observed in both control (+0.60 total relative abundance, *p* < 1e-7) and Ang II (+0.63 total relative abundance, *p* < 1e-7) groups indicated the predominance of temperate viruses in the murine gut (Figure 3b). In contrast, the total relative abundance of temperate viruses was significantly lower in VLP metagenomes from both control (0.82 cumulative relative abundance, *p* < 1e-7) and Ang II (−0.71, *p* < 1e-6) groups (Figure 3b), suggesting that temperate phages are predominantly maintained as prophages in the murine gut.

### Effect of diet on the gut microbiota and hypertension

**Figure 4.**
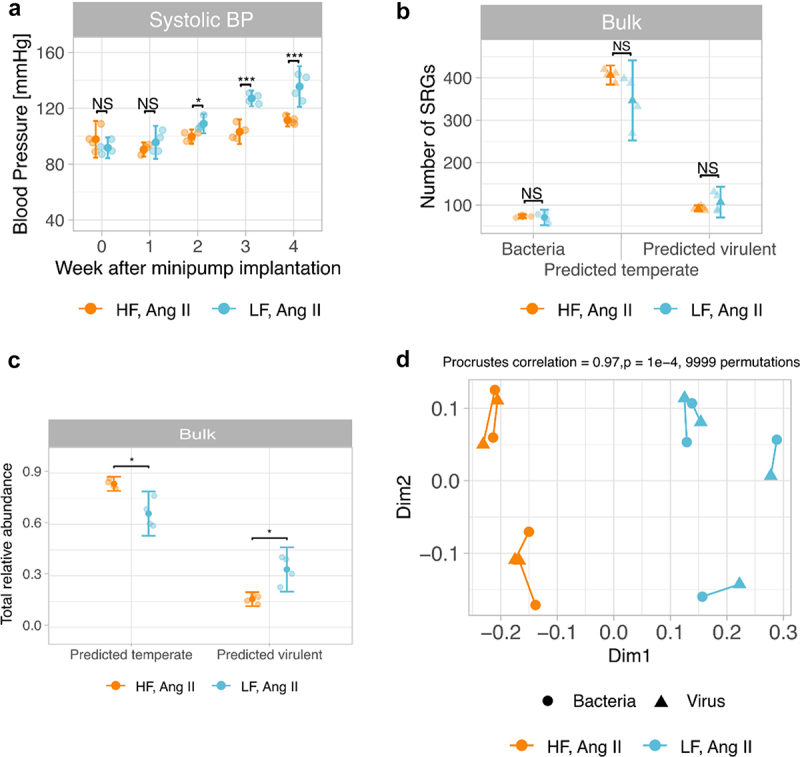
High-fiber diet prevented hypertension development and altered bacterial and viral populations and functional bacterial profiles in murine gut. After four weeks of implantation of a minipump with Ang II, a, blood pressure (BP) of high-fiber-fed Ang II mice was lower compared to low-fiber-fed Ang II mice. High fiber did not change b, the richness of gut bacterial and viral populations. However, bacterial and viral composition changed between diets. c, Procrustes rotation of the viral community PCoA coordinates from bulk metagenomes versus bacterial community PCoA coordinates; Bray Curtis metric was used in both cases. N = 4 mice/group. *p<.05; **p<.01; ***p<.001; NS p ≥ 0.05. Two-way ANOVA with Tukey post hoc test (a), two-pair group Wilcoxon-test (b). The horizontal lines represent the median. (a, b), and each dot represents the measure of each mouse (a-c).

To investigate the impact of a diet rich in fermentable fiber on the gut viral and bacterial populations in the Ang II model of hypertension, six-week-old male C57BL/6J mice were fed either a high-fiber or a low-fiber diet for 4 weeks after the implantation of a minipump containing Ang II. Consistent with previous studies,^9,30^ we observed that high-fiber-fed mice had significantly lower systolic BP (−24 mmHg, *p* = .00005) compared to low-fiber-fed mice 4 weeks after implanting the minipump (Figure 4a). This confirms that a diet rich in fermentable fiber can prevent the development of hypertension in mice treated with Ang II.

We further examined the impact of fermentable fiber on the gut bacterial and viral communities. We observed some bacterial and viral community differences between mice (Supplementary Figure 4a and Supplementary Figure 5a); however, these communities were more similar between mice fed with the same diet than mice fed with different diets (Supplementary Figure 4b and Supplementary Figure 5b). Indeed, diet contributed to 47.7% of the variance of the bacterial populations (*p* = .029, PERMANOVA analysis using Bray-Curtis distances and 9,999 permutations) and 40.7% of the variance of viral populations (*p* = .029, PERMANOVA analysis using Bray-Curtis distances and 9,999 permutations). These changes were not detected in terms of observed bacterial (*p* = 1) and viral (*p* = .8857) SRGs, which did not significantly differ between high-fiber- and low-fiber-fed mice (Figure 4b), nor did the ACE, Chao1, Simpson, or Shannon indexes (Supplementary Figure 6).

As we observed before, predicted temperate viruses outnumber predicted virulent viruses in both treatments (Figure 4b). However, the total relative abundance of temperate viruses was significantly higher in high-fiber-fed mice than in low-fiber-fed mice (Figure 4c). In addition, we observed a strong correlation between the composition of viral and bacterial communities (*r* = 0.96, *p* = 5e-4, Figure 4d) using a Procrustes rotation of the bacterial community PCoA coordinates versus viral community PCoA coordinates. These results indicate that gut populations of viruses, including temperate and virulent, and bacteria change with diet, and the changes in these viral and bacterial populations mirror or shape each other.

**Figure 5.**
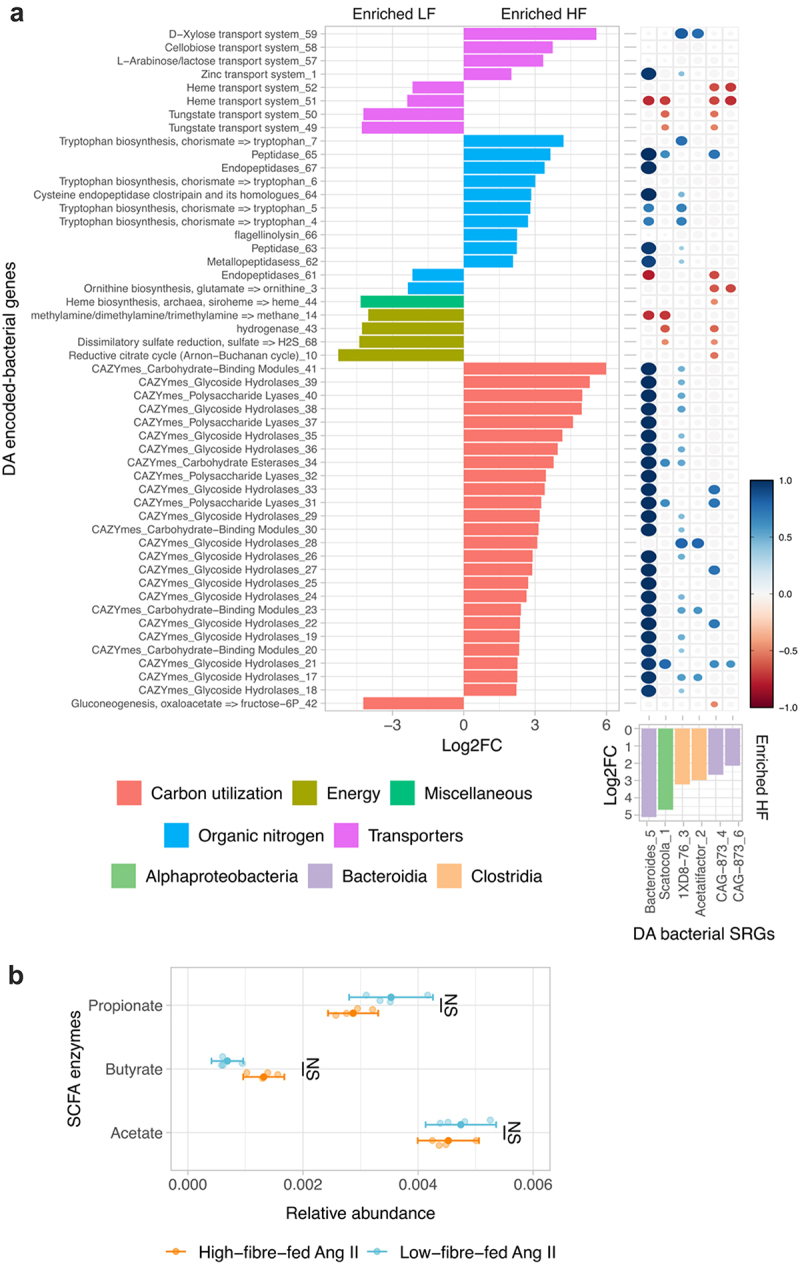
Differentially abundant (DA) genes between bacterial populations of high fiber- and low fiber-fed mice. a, DA genes (left panel) and bacteria (bottom right panel) and their correlation (top right panel) are presented. In the left panel, the log2 fold change in mean abundance of genes that are differently abundant in the bacterial community of high fiber-fed mice compared to low fiber-fed mice are shown. Positive coefficient indicates the enrichment in high-fiber-fed-mice gut bacterial community. The bar color represents DRAM-v functional categories of genes. Similarly, the log2 fold change in mean abundance of DA bacterial SRGs of high-fiber-fed mice compared to low-fiber-fed mice is presented in the bottom right panel color based on bacterial phylogenetic class. Spearman correlation coefficients of only significant correlations between DA genes and DA bacterial SRGs are presented in the top right panel. B, cumulative relative abundances of enzymes associated with the production of acetate, butyrate, and propionate. *p < .05; **p < .01; ***p < .001; NS p ≥ 0.05. Consensus differential features with | log2FC |>2 from DeSeq, MaAsLin, and metagenomeSeq outputs using dar (a).^50^ Spearman correlation with FDR corrections for multiple testing (a), pairwise t-test with FDR corrections for multiple testing (b).

To explore the main differences in the gut composition of bacteria, their genes, and viruses between high fiber and low fiber diets, we detected differentially abundant (DA) SRGs or genes. We only considered DA features when they were found across three different methods (see Methods). The microbiome of high fiber-fed mice was enriched in six bacterial SRGs, 42 viral SRGs, and 39 bacterial-encoded genes (see summary statistics in Supplementary Tables 2, 3 and 4, as well as in Supplementary Figures 7, 8, and 9, respectively). Twenty-five of these high-fiber enriched genes encode carbohydrate-active enzymes (CAZymes), which are employed to break down fiber glycans into glucose, and the remaining 14 genes were associated with transport systems of D-xylose, cellobiose, L-arabinose, and lactose, and tryptophan metabolism (Figure 5a). Tryptophan is the precursor to indole-3-propionic acid, which has shown protection against the development of heart failure in a model of heart failure with preserved ejection fraction.^51^ Furthermore, the enriched CAZyme-family GH43 was the most abundant DA gene in high-fiber fed mice. GH43 CAZymes degrade arabinan, a plant polysaccharide. The higher abundance of CAZymes was significantly correlated with the abundance of *Bacteroides caecimuris* (Bacteroides_5 in Figure 5a), which was either the most abundant or the second most abundant bacterial SRG within the gut of high fiber fed mice and encoded 28 GH43 CAZymes. Additionally, the six enriched bacteria in high fiber encoded enzymes to produce acetate and five encoded enzymes to produce butyrate. Acetate and butyrate are two out of the three most predominant SCFAs. SCFAs lower BP in both experimental and human hypertension.^9,52,53^ However, the relative abundance of SCFA-associated enzymes was not significantly different between diet groups (Figure 5b). The microbiome of low fiber-fed mice, in contrast, was enriched in 34 viral SRGs and 12 bacterial-encoded genes associated with the biosynthesis of ornithine, ubiquinone, and heme biosynthesis, as well as enzymes associated with the production of gases, such as methane and hydrogen sulfide (H_2_S), and the transport of heme, and tungstate (Figure 5a).

Delving into the composition and abundance of the 76 DA viral SRGs (Supplementary Figures 8 and 10), we observed four main results. First, the proportion of DA viral SRGs that were predicted as temperate was higher in high fiber mice (88.1%) compared to low fiber mice (41.2%). Second, 24 out of the 76 DA viral SRGs encoded auxiliary metabolic genes (AMGs). By expressing these genes, viruses might modulate their bacterial host metabolism.^54,55^ Twenty of the 24 SRGs-encoding AMGs (83.3%) were enriched in high-fiber-fed mice, and 18 were predicted to be temperate viruses. Third, 9 out of the 76 DA viral SRGs had a relative abundance higher than 0.01 in at least two mice from the same treatment (Supplementary Figure 8). Five of these 9 viral SRGs were predicted as temperate viruses and were enriched in high-fiber-fed mice, while the remaining four were predicted as virulent viruses and were enriched in low-fiber-fed mice. Fourth, the 5 most abundant temperate viral SRGs were identified as prophages within either *Bacteroides caecimuris_5* (4 of them) or *Scatocola sp910577205_1* (1 of them) DA bacteria. The host of the 4 virulent viral SRGs was not determined. The relative abundance of the 5 most abundant temperate DA viral SRGs significantly correlated with the abundance of their host, which also was differently enriched in high fiber-fed mice (Supplementary Figure 9), suggesting that these SRGs are replicating mainly through lysogenic cycles.

## Discussion

To investigate the link between gut bacterial and viral populations, diet, and experimental hypertension, we assembled a collection of 106 bacterial and 816 viral, including 580 temperate and 236 virulent phages, single-species representative genomes from the murine gut. Based on this collection, we explored alterations in the gut microbiome in Ang II-driven hypertension. We observed that Ang II-challenged mice fed with a control diet presented a higher systolic BP than Sham-control mice fed with a control diet, but we found no differences in gut viral and bacterial between Ang II-challenged and Sham-control mice. By comparing VLPs and bulk metagenomes, we observed that gut temperate viruses, which were found in low abundance as virions, outnumbered virulent viruses in both Sham-control and Ang II mouse samples. Furthermore, we found that the gut microbiome, in terms of bacterial, bacterial-encoded genes, and viruses, significantly varied between high-fiber-fed and low-fiber-fed Ang II-challenged mice, showing complex diet–microbiome interactions that may affect BP regulation.

We did not observe an effect of Ang II-driven hypertension on the murine gut microbiome. This result is consistent with a previous study, which analyzed more than 500 murine samples using 16S rRNA data and found that Ang II contributed to only a small percentage (0.4%) of the variance in the microbiome.^56^ This result, however, does not exclude that the gut microbiome contributes to different aspects of BP regulation.^4^ Previous studies have shown that germ-free mice transplanted with fecal samples from hypertensive patients developed an increased BP.^5^ Moreover, phenotypic responses of germ-free mice challenged with Ang II, in terms of organ damage and circulating metabolites, varied from the responses of either colonized or conventionalized Ang II-challenged mice, suggesting a role for the gut microbiome in the phenotypic response to a hypertensive stimulus.^57,58^ Thus, factors affecting the status of the gut microbiome and the gut microbiome *per se* may affect the development of hypertension.

Similar to previous studies, a diet rich in fiber prevented the development of hypertension in a mouse model.^9,30^ This protective phenotype has been previously linked to increased production of gut microbial-derived SCFAs,^53,59^ derived from microbial degradation of dietary fiber.^59,60^ In this study, we did not observe significant differences in the abundance of enzymes involved in the synthesis of SCFAs across diets. We found instead an enrichment on the abundance of several CAZymes, especially the GH43 family, which degrade plant polysaccharides, coupled with a higher abundance of six bacterial SRGs that encode CAZymes and enzymes involved in the synthesis of the SCFAs acetate and butyrate. In contrast, the microbiome of low fiber-fed mice was enriched with genes associated with the metabolism of hydrogen to produce H_2_S and methane. Gut microbes that utilize hydrogen mainly fall into three functional groups: methanogens, sulfate-reducing bacteria (SRB), and acetogens.^61^ Higher levels of SRB accompanied by higher concentrations of H_2_S and lower levels of SCFAs have been observed in mice consuming a diet rich in fat and simple sugars compared to mice consuming a diet low in fat and high in plant polysaccharides.^62^ Therefore, we hypothesize that in the same way that has been observed in sequential batch fecal fermentations *in vitro*, fiber drives the selection of gut bacteria encoding CAZymes, which then determine the availability of substrates to cross-fed other bacterial members and the production of secondary metabolism such as SCFA and H_2_S.^63^ If correct, it indicates the relevance of characterizing gut bacterial populations that encode CAZymes as a criterion to design and monitor the efficiency of personalized diets as a therapy tool for hypertension.

Fiber intake in the Ang II mouse model affected the murine gut virome, which was consistently composed of a higher number of temperate viruses. Compared to the gut virome of low-fiber-fed mice (reconstructed from bulk metagenomes), the virome of high-fiber mice harbored a higher total relative abundance of temperate viruses. Aligned with this, temperate viruses comprised 88.1% of the enriched viral SRGs in high-fiber-fed mice, while they only comprised 41.2% of the enriched viral SRGs in low-fiber-fed mice. Indeed, the five most prevalent enriched viral SRGs in high-fiber-fed mice were found as prophages of two out of the six enriched bacterial SRGs. Together, these results match with upcoming evidence that gut viruses follow a ‘*piggy-back-the-winner’* dynamic, in which lysogeny predominates at high microbial abundance,^64^ and raise the question of the role of prophages in providing a competitive advantage to their bacterial host. This competitive advantage may include a variety of strategies that have been previously observed, such as the expression of auxiliary metabolic genes (predominantly found in DA viral SRGs of high-fiber mice), the infection of a competitor strain, prophage-driven horizontal gene transfer and superinfection exclusion.^16,65–67^ These observations, along with the strong correlation between gut viral and bacterial populations, leave open the question of whether phages are mirroring or shaping the gut microbiome.^68^ To answer this question is crucial to better understand how prophages affect the fitness of their bacterial host and how dietary fibers impact these phage-bacteria interactions. This knowledge is essential for leveraging prophages as therapeutic tools coupled with diet to prevent and treat hypertension.

As limitations, we acknowledge the mice sample size per experiment, the use of exclusively male C57BL/6J mice, and the lack of robust methodologies to predict phages lifecycles and to discriminate low abundance temperate phages from bacterial contamination in VLP metagenomes. Moreover, the lack of changes in the microbiome of Ang II-driven hypertension compared to Sham-control suggests that the use of other experimental models could be more suitable to study the link between the development of hypertension (such as the spontaneously hypertensive rat) and the gut microbiome, as well as similar approaches in human samples. This study, however, provides a robust methodology to comprehensively reconstruct gut viral and bacterial populations in hypertension in any of these models and present novel hypotheses to build upon and expand our understanding of gut phages and hypertension.

In conclusion, this study combined bulk and VLP metagenomes to study gut viral populations and their lifecycles, alongside bacterial populations in experimental hypertension. Using this methodology, we observed an intriguing pattern of temperate and virulent viruses in the gut and characterized the virome and bacteriome of the Ang II hypertensive model. We found no differences in the gut virome and bacteriome of Ang II-driven hypertensive and Sham-control mice fed a control diet. However, a high-fiber diet rescued the induction of hypertension in Ang II treated mice, and this response was accompanied by changes between gut bacterial and viral communities, and the bacteria gene-repertoire likely associated with cross-feeding of carbohydrate fermentation products, SCFAs, and hydrogen. These show complex interactions between diet, gut bacteria and phages that may be relevant to BP regulation and may represent a new therapeutic opportunity to treat hypertension, particularly when dietary interventions are involved.

## Supplementary Material

Supplemental Material

## Data Availability

Supplementary tables and figures are available at Figshare DOI: https://doi.org/10.26180/25366162.v1. The raw sequencing data for this study has been deposited in the European Nucleotide Archive (ENA) at EMBL-EBI under accession number PRJEB73709.
