# Supplementary material for: Dietary fiber intake impacts gut bacterial and viral populations in a hypertensive mouse model
Source: Gut Microbes. 2024 Sep 28;16(1):2407047. doi: 10.1080/19490976.2024.2407047 (PMC11567275; doi:10.1080/19490976.2024.2407047)
Supplement: Supplemental Material [file KGMI_A_2407047_SM5499.zip › Online supplemental file Aug 16th.docx]

**Online supplemental file to**

**Dietary fibre intake impacts gut bacterial and viral populations in a hypertensive mouse model**

Laura Avellaneda-Franco,^1^ Liang Xie,^1,2^ Michael Nakai,^1^ Jeremy J. Barr,^1^* Francine Z. Marques^1,3,4^*

^1^School of Biological Sciences, Monash University, Melbourne, Australia; ^2^ Precision Medicine Translational Research Programme, Department of Obstetrics & Gynaecology, Yong Loo Lin School of Medicine, National University of Singapore, Singapore; ^3^Heart Failure Research Group, Baker Heart and Diabetes Institute, Melbourne, Australia; ^4^Victorian Heart Institute, Monash University, Melbourne, Australia.

*Co-senior authors.

**Correspondence to**: Professor Francine Marques. 25 Rainforest Walk, Clayton, Monash University, VIC Australia 3800. E: francine.marques@monash.edu

**Supplementary Tables**

**Supplementary Table 1. Summary statistics of the relative abundance of each Distilled and Refined Annotation of Metabolism (DRAM) gene functional category across angiotensin II (Ang II) and saline (Sham) treatments.**

| **Mouse** | **Treatment** | **Functional DRAM category** | **Relative abundance** | **Lower limit  95% CI** | **Upper limit  95% CI** | **Mean per treatment** |
| --- | --- | --- | --- | --- | --- | --- |
| M1 | Sham | carbon_utilization | 0.2629 | 0.2404 | 0.2702 | 0.2553 |
| M2 | Sham | carbon_utilization | 0.2428 | 0.2404 | 0.2702 | 0.2553 |
| M3 | Sham | carbon_utilization | 0.2536 | 0.2404 | 0.2702 | 0.2553 |
| M4 | Sham | carbon_utilization | 0.2621 | 0.2404 | 0.2702 | 0.2553 |
| M5 | Ang II | carbon_utilization | 0.2448 | 0.2362 | 0.2685 | 0.2524 |
| M6 | Ang II | carbon_utilization | 0.2427 | 0.2362 | 0.2685 | 0.2524 |
| M7 | Ang II | carbon_utilization | 0.2631 | 0.2362 | 0.2685 | 0.2524 |
| M8 | Ang II | carbon_utilization | 0.2589 | 0.2362 | 0.2685 | 0.2524 |
| M1 | Sham | carbon_utilization_woodcroft | 0.0211 | 0.0192 | 0.0234 | 0.0213 |
| M2 | Sham | carbon_utilization_woodcroft | 0.0212 | 0.0192 | 0.0234 | 0.0213 |
| M3 | Sham | carbon_utilization_woodcroft | 0.0230 | 0.0192 | 0.0234 | 0.0213 |
| M4 | Sham | carbon_utilization_woodcroft | 0.0198 | 0.0192 | 0.0234 | 0.0213 |
| M5 | Ang II | carbon_utilization_woodcroft | 0.0186 | 0.0182 | 0.0196 | 0.0189 |
| M6 | Ang II | carbon_utilization_woodcroft | 0.0193 | 0.0182 | 0.0196 | 0.0189 |
| M7 | Ang II | carbon_utilization_woodcroft | 0.0184 | 0.0182 | 0.0196 | 0.0189 |
| M8 | Ang II | carbon_utilization_woodcroft | 0.0192 | 0.0182 | 0.0196 | 0.0189 |
| M1 | Sham | energy | 0.0798 | 0.0771 | 0.0817 | 0.0794 |
| M2 | Sham | energy | 0.0812 | 0.0771 | 0.0817 | 0.0794 |
| M3 | Sham | energy | 0.0780 | 0.0771 | 0.0817 | 0.0794 |
| M4 | Sham | energy | 0.0785 | 0.0771 | 0.0817 | 0.0794 |
| M5 | Ang II | energy | 0.0805 | 0.0791 | 0.0818 | 0.0805 |
| M6 | Ang II | energy | 0.0796 | 0.0791 | 0.0818 | 0.0805 |
| M7 | Ang II | energy | 0.0816 | 0.0791 | 0.0818 | 0.0805 |
| M8 | Ang II | energy | 0.0802 | 0.0791 | 0.0818 | 0.0805 |
| M1 | Sham | MISC | 0.2263 | 0.2117 | 0.2351 | 0.2234 |
| M2 | Sham | MISC | 0.2124 | 0.2117 | 0.2351 | 0.2234 |
| M3 | Sham | MISC | 0.2266 | 0.2117 | 0.2351 | 0.2234 |
| M4 | Sham | MISC | 0.2283 | 0.2117 | 0.2351 | 0.2234 |
| M5 | Ang II | MISC | 0.2358 | 0.2000 | 0.2491 | 0.2245 |
| M6 | Ang II | MISC | 0.2369 | 0.2000 | 0.2491 | 0.2245 |
| M7 | Ang II | MISC | 0.2215 | 0.2000 | 0.2491 | 0.2245 |
| M8 | Ang II | MISC | 0.2040 | 0.2000 | 0.2491 | 0.2245 |
| M1 | Sham | organic_nitrogren | 0.2849 | 0.2800 | 0.2909 | 0.2855 |
| M2 | Sham | organic_nitrogren | 0.2875 | 0.2800 | 0.2909 | 0.2855 |
| M3 | Sham | organic_nitrogren | 0.2809 | 0.2800 | 0.2909 | 0.2855 |
| M4 | Sham | organic_nitrogren | 0.2886 | 0.2800 | 0.2909 | 0.2855 |
| M5 | Ang II | organic_nitrogren | 0.2933 | 0.2784 | 0.2974 | 0.2879 |
| M6 | Ang II | organic_nitrogren | 0.2826 | 0.2784 | 0.2974 | 0.2879 |
| M7 | Ang II | organic_nitrogren | 0.2928 | 0.2784 | 0.2974 | 0.2879 |
| M8 | Ang II | organic_nitrogren | 0.2829 | 0.2784 | 0.2974 | 0.2879 |
| M1 | Sham | transporters | 0.1251 | 0.1117 | 0.1586 | 0.1351 |
| M2 | Sham | transporters | 0.1549 | 0.1117 | 0.1586 | 0.1351 |
| M3 | Sham | transporters | 0.1379 | 0.1117 | 0.1586 | 0.1351 |
| M4 | Sham | transporters | 0.1227 | 0.1117 | 0.1586 | 0.1351 |
| M5 | Ang II | transporters | 0.1269 | 0.1127 | 0.1590 | 0.1359 |
| M6 | Ang II | transporters | 0.1390 | 0.1127 | 0.1590 | 0.1359 |
| M7 | Ang II | transporters | 0.1225 | 0.1127 | 0.1590 | 0.1359 |
| M8 | Ang II | transporters | 0.1550 | 0.1127 | 0.1590 | 0.1359 |

The relative abundance per mouse is presented along with the mean, lower, and upper limits of 95% confidence interval. Legend: Ang II, angiotensin II; CI, confidence interval.

**Supplementary Table 2. Summary statistics of differentially abundant (DA) bacterial species-level representative genomes (SRGs) across high fibre-fed mice and low fibre-fed mice.**

| **Bacteria SRG** | **Class** | **Order** | **Family** | **Genus** | **Species** | **Strain** | **Figure axis label** | **P-value adjsuted** | **log2FC** |
| --- | --- | --- | --- | --- | --- | --- | --- | --- | --- |
| L2_bin.55 | Alphaproteobacteria | RF32 | CAG-239 | Scatocola | Scatocola sp910577205 | Scatocola sp910577205_16 | Scatocola_1 | 5.31E-06 | 4.6884 |
| L3_bin.16 | Clostridia | Lachnospira-les | Lachnospira-ceae | Acetatifactor | Acetatifactor sp910576125 | Acetatifactor sp910576125_18 | Acetatifactor_2 | 5.16E-03 | 2.9720 |
| M4_bin.50 | Clostridia | Lachnospirales | Lachnos-piraceae | 1XD8-76 | 1XD8-76 sp910573755 | 1XD8-76 sp910573755_80 | 1XD8-76_3 | 9.76E-04 | 3.2286 |
| M4_bin.93 | Bacteroidia | Bacteroidales | Muribacu-laceae | CAG-873 | CAG-873 sp009775225 | CAG-873 sp009775225_87 | CAG-873_4 | 1.48E-02 | 2.6696 |
| M5_bin.21 | Bacteroidia | Bacteroidales | Bacteroi-daceae | Bacteroides | Bacteroides caecimuris | Bacteroides caecimuris_91 | Bacteroides_5 | 2.80E-08 | 5.1129 |
| M6_bin.18 | Bacteroidia | Bacteroidales | Muribacu-laceae | CAG-873 | CAG-873 sp002490635 | CAG-873 sp002490635_95 | CAG-873_6 | 2.48E-02 | 2.1488 |

Each bacterial SRG and its phylogenetic information is presented. Additionally, the base mean, log2 fold change (FC) in the mean abundance of high fibre-fed mice compared to low fibre-fed mice, and FDR adjusted p-values are presented per each DA bacterial SRG. N=4 mice/group.

**Supplementary Table 3. Summary statistics of differentially abundant (DA) viral species-level representative genomes (SRGs) across high fibre-fed mice and low fibre-fed mice.**

| **Phage** | **log2FC** | **P-value adjsuted** | **Family** | **Temperate** | **encoding AMG** |
| --- | --- | --- | --- | --- | --- |
| unbinned_NODE_1021_length_39956_cov_46.432696 | 2.663896422 | 0.023535873 | Siphoviridae | Yes | Yes |
| unbinned_NODE_104_length_138764_cov_41.745669_1 | 3.758252459 | 0.007311803 | Siphoviridae | Yes | Yes |
| unbinned_NODE_109_length_126434_cov_28.307037_1 | 3.178192425 | 0.019361531 | Siphoviridae | Yes | Yes |
| unbinned_NODE_1102_length_19992_cov_25.645734 | -4.903191432 | 0.000985419 | Siphoviridae | No | No |
| unbinned_NODE_1350_length_15592_cov_179.301281 | -3.124198755 | 0.003322585 | Podoviridae | No | No |
| unbinned_NODE_1374_length_15413_cov_52.934510 | -3.429225882 | 0.021428359 | Podoviridae | No | No |
| unbinned_NODE_1521_length_16515_cov_7.326428 | 3.34944442 | 0.003108793 | Podoviridae | No | No |
| unbinned_NODE_1750_length_16581_cov_17.198657 | 3.412791851 | 0.000763895 | Not assigned | No | No |
| unbinned_NODE_1821_length_24122_cov_177.416088 | 2.557548194 | 0.01524765 | Siphoviridae | No | Yes |
| unbinned_NODE_203_length_91359_cov_26.073622_1 | -2.463948816 | 0.036976881 | Siphoviridae | Yes | No |
| unbinned_NODE_2178_length_5696_cov_128.776635 | -7.267968702 | 7.00E-07 | Microviridae | No | No |
| unbinned_NODE_2296_length_16533_cov_78.339726 | 3.391659033 | 0.002781162 | Podoviridae | No | No |
| unbinned_NODE_247_length_63952_cov_61.585990 | 3.336985393 | 0.010364333 | Siphoviridae | Yes | Yes |
| unbinned_NODE_251_length_35162_cov_16.630843 | -2.556357632 | 0.021428359 | Siphoviridae | No | Yes |
| unbinned_NODE_302_length_59635_cov_20.078483 | 3.74095888 | 0.006191589 | Siphoviridae | Yes | No |
| unbinned_NODE_365_length_56519_cov_135.820806 | 3.581773373 | 0.006956367 | Siphoviridae | Yes | Yes |
| unbinned_NODE_373_length_50632_cov_32.054412 | 2.745333011 | 0.022196632 | Siphoviridae | Yes | Yes |
| unbinned_NODE_390_length_39183_cov_25.216137 | -4.19543719 | 0.004081441 | Not assigned | Yes | No |
| unbinned_NODE_392_length_50653_cov_8.306613 | 2.743987969 | 0.03632761 | Siphoviridae | Yes | Yes |
| unbinned_NODE_394_length_35159_cov_26.193112 | -3.673093028 | 0.008130096 | Siphoviridae | No | No |
| unbinned_NODE_442_length_50643_cov_47.005673 | 3.358887615 | 0.01524765 | Siphoviridae | Yes | Yes |
| unbinned_NODE_442_length_50795_cov_11.971659 | 3.358887615 | 0.01524765 | Siphoviridae | Yes | Yes |
| unbinned_NODE_456_length_41330_cov_15.728407 | -4.35131274 | 0.001968789 | Siphoviridae | No | Yes |
| unbinned_NODE_465_length_40594_cov_27.952860_1 | -3.410024479 | 0.009614703 | Siphoviridae | Yes | Yes |
| unbinned_NODE_471_length_40130_cov_617.176519 | 3.276353959 | 0.001191938 | Siphoviridae | Yes | No |
| unbinned_NODE_510_length_37407_cov_299.616915 | 5.369768976 | 0.000334059 | Not assigned | Yes | No |
| unbinned_NODE_524_length_21152_cov_92.035455 | -5.824718894 | 6.80E-05 | Podoviridae | No | No |
| unbinned_NODE_533_length_36311_cov_11.558390 | -2.453141417 | 0.033310148 | Myoviridae | Yes | Yes |
| unbinned_NODE_555_length_37891_cov_7.122238 | -2.410192027 | 0.042149104 | Siphoviridae | No | No |
| unbinned_NODE_555_length_40400_cov_51.666700 | 4.632088139 | 0.001191938 | Not assigned | Yes | No |
| unbinned_NODE_592_length_43088_cov_22.492064_1 | -2.473014827 | 0.031131322 | Myoviridae | Yes | Yes |
| unbinned_NODE_639_length_31410_cov_22.123043 | -2.39918064 | 0.023205131 | Siphoviridae | No | Yes |
| unbinned_NODE_648_length_28858_cov_6.271847 | -3.387843859 | 0.01524765 | Siphoviridae | No | No |
| unbinned_NODE_650_length_40551_cov_5.663967 | 3.309192069 | 0.017514901 | Siphoviridae | Yes | No |
| unbinned_NODE_652_length_45514_cov_44.174311 | 2.905107066 | 0.025163264 | Siphoviridae | Yes | Yes |
| unbinned_NODE_662_length_39904_cov_7.186379 | 2.770295275 | 0.018948213 | Siphoviridae | Yes | Yes |
| unbinned_NODE_688_length_37040_cov_114.582777 | 5.434218649 | 0.000244441 | Not assigned | Yes | No |
| unbinned_NODE_708_length_33039_cov_88.252456 | 4.491077696 | 0.001191938 | Siphoviridae | Yes | Yes |
| unbinned_NODE_719_length_51057_cov_39.074958 | -2.44416248 | 0.037839754 | Siphoviridae | Yes | No |
| unbinned_NODE_728_length_16378_cov_21.610550 | -4.371574732 | 0.00320164 | Podoviridae | No | No |
| unbinned_NODE_734_length_39126_cov_16.308976 | -4.15945925 | 0.00445613 | Not assigned | Yes | No |
| unbinned_NODE_745_length_35223_cov_367.562898 | 5.045212686 | 0.00094456 | Siphoviridae | Yes | No |
| unbinned_NODE_749_length_35059_cov_33.981574 | 2.906843798 | 0.016479338 | Siphoviridae | No | Yes |
| unbinned_NODE_750_length_35168_cov_75.963432 | 5.045471457 | 0.00094456 | Siphoviridae | Yes | No |
| unbinned_NODE_756_length_35690_cov_8.502315 | -3.872287163 | 0.007330351 | Siphoviridae | Yes | No |
| unbinned_NODE_800_length_28468_cov_9.601133 | -2.393319655 | 0.027274154 | Siphoviridae | Yes | No |
| unbinned_NODE_863_length_14053_cov_8.880422 | -4.400752758 | 0.003370167 | Not assigned | No | No |
| unbinned_NODE_901_length_23173_cov_12.928584 | -2.300061586 | 0.02873258 | Myoviridae | No | No |
| virome_NODE_112_length_15595_cov_73.439704 | -3.083068043 | 0.003082724 | Podoviridae | No | No |
| virome_NODE_12_length_58902_cov_363.806362 | 3.464500353 | 0.008646701 | Siphoviridae | Yes | Yes |
| virome_NODE_13_length_58792_cov_35.512113 | 3.226296291 | 0.011093126 | Siphoviridae | Yes | Yes |
| virome_NODE_143_length_21188_cov_3563.957318 | -5.767643909 | 3.85E-05 | Podoviridae | No | No |
| virome_NODE_16_length_58855_cov_109.807891 | 3.18082217 | 0.011907668 | Siphoviridae | Yes | Yes |
| virome_NODE_17_length_58652_cov_16.242504 | 3.11584785 | 0.019291469 | Siphoviridae | Yes | Yes |
| virome_NODE_22_length_58599_cov_1020.723934 | 3.177798355 | 0.016096239 | Siphoviridae | Yes | Yes |
| virome_NODE_284_length_15600_cov_1543.733419 | -2.945203929 | 0.005218403 | Podoviridae | No | No |
| virome_NODE_32_length_41185_cov_65.104109 | 3.25249314 | 0.001191938 | Siphoviridae | Yes | No |
| virome_NODE_42_length_35057_cov_20.424033 | 5.002729494 | 0.000985419 | Siphoviridae | Yes | No |
| virome_NODE_42_length_40390_cov_21.632428 | 4.632687056 | 0.001191938 | Not assigned | Yes | No |
| virome_NODE_45_length_33933_cov_15.388069 | 4.518235014 | 0.00064301 | Myoviridae | Yes | No |
| virome_NODE_46_length_40703_cov_103.662394 | 3.30247353 | 0.001191938 | Siphoviridae | Yes | No |
| virome_NODE_47_length_40744_cov_47.423972 | 3.328639014 | 0.001105668 | Siphoviridae | Yes | No |
| virome_NODE_51_length_21286_cov_257.433753 | -5.71719799 | 6.80E-05 | Podoviridae | No | No |
| virome_NODE_51_length_39092_cov_61.742279 | 4.60808078 | 0.001191938 | Not assigned | Yes | No |
| virome_NODE_53_length_33966_cov_35.629324 | -2.443552963 | 0.035547584 | Myoviridae | Yes | Yes |
| virome_NODE_56_length_40390_cov_19.087393 | 4.619927219 | 0.001191938 | Not assigned | Yes | No |
| virome_NODE_6_length_59652_cov_1379.063443 | 3.757652115 | 0.006127755 | Siphoviridae | Yes | No |
| virome_NODE_61_length_35277_cov_101.108966 | -2.433539997 | 0.046405068 | Siphoviridae | Yes | No |
| virome_NODE_62_length_33848_cov_22.496079 | -2.457235255 | 0.035431095 | Myoviridae | Yes | Yes |
| virome_NODE_69_length_31916_cov_149.151722 | 3.108337318 | 0.0145849 | Siphoviridae | Yes | Yes |
| virome_NODE_70_length_35682_cov_149.092654 | -3.872287163 | 0.007330351 | Siphoviridae | Yes | No |
| virome_NODE_71_length_21249_cov_1376.137397 | -5.581458559 | 9.92E-05 | Podoviridae | No | No |
| virome_NODE_74_length_38615_cov_19.140197 | 4.834646634 | 3.58E-05 | Not assigned | Yes | No |
| virome_NODE_79_length_21206_cov_1323.918964 | -5.736251632 | 6.80E-05 | Podoviridae | No | No |
| virome_NODE_94_length_34010_cov_33.420851 | 4.855733212 | 0.000389629 | Myoviridae | Yes | No |
| virome_NODE_98_length_33752_cov_19.040182 | -2.365596854 | 0.038788554 | Myoviridae | Yes | Yes |

Additional information regarding whether the DA viral SRG was catalogued as temperate and encode auxiliary metabolic genes (AMGs) is shown. The log2 fold change (FC) in the mean abundance of high fibre-fed mice compared to low fibre-fed mice, and FDR adjusted p-values are presented per each viral VC. N=4 mice/group.

**Supplementary Table 4. Summary statistics of differentially abundant (DA) genes across high fibre-fed mice and low fibre-fed mice.**

| **Gene description** | **Figure axis label** | **Functional DRAM category** | **Functional DRAM subcategory** | **KEGG module** | **Adjusted p-value** | **log2FC** |
| --- | --- | --- | --- | --- | --- | --- |
| xylF; D-xylose transport system substrate-binding protein | D-Xylose transport system_59 | transporters | Transporters | D-Xylose transport system | 1.67E-07 | 5.579545063 |
| cebF; cellobiose transport system permease protein | Cellobiose transport system_58 | transporters | Transporters | Cellobiose transport system | 0.002056889 | 3.741776978 |
| lacF; lactose/L-arabinose transport system permease protein | L-Arabinose/lactose transport system_57 | transporters | Transporters | L-Arabinose/lactose transport system | 0.003431334 | 3.339871978 |
| NA | Zinc transport system_1 | transporters | Transporters | NA | 0.000442926 | 2.00408084 |
| ccmC; heme exporter membrane protein CcmC | Heme transport system_52 | transporters | Transporters | Heme transport system | 0.002921607 | -2.154072309 |
| ccmB; heme exporter membrane protein CcmB | Heme transport system_51 | transporters | Transporters | Heme transport system | 0.000740018 | -2.376870765 |
| tupB; tungstate transport system permease protein | Tungstate transport system_50 | transporters | Transporters | Tungstate transport system | 7.61E-10 | -4.223805032 |
| tupA; tungstate transport system substrate-binding protein | Tungstate transport system_49 | transporters | Transporters | Tungstate transport system | 2.62E-08 | -4.287258543 |
| indole-3-glycerol phosphate synthase [EC:4.1.1.48] [RN:R03508] | Tryptophan biosynthesis, chorismate => tryptophan_7 | organic_nitrogren | Amino Acid | Tryptophan biosynthesis, chorismate => tryptophan | 6.35E-21 | 4.195472305 |
| Catlytic type: Metallo; a member is EcxAB peptidase (Escherichia coli) | Peptidase_65 | organic_nitrogren | Peptidase | Peptidase | 1.21E-10 | 3.647002232 |
| Catlytic type: Metallo; inactivates the anti-sigma factor RsiW by performing the first of several cleavages | Endopeptidases_67 | organic_nitrogren | Peptidase | Endopeptidases | 0.000202387 | 3.401063412 |
| phosphoribosylanthranilate isomerase [EC:5.3.1.24] [RN:R03509] | Tryptophan biosynthesis, chorismate => tryptophan_6 | organic_nitrogren | Amino Acid | Tryptophan biosynthesis, chorismate => tryptophan | 9.59E-16 | 3.009552669 |
| Catlytic type: Cysteine; Selective for hydrolysis of arginyl bonds; It requires calcium ions for activity, as well as a reducing environment; member is clostripain | Cysteine endopeptidase clostripain and its homologues_64 | organic_nitrogren | Peptidase | Cysteine endopeptidase clostripain and its homologues | 2.69E-05 | 2.834641944 |
| anthranilate phosphoribosyltransferase [EC:2.4.2.18] [RN:R01073] | Tryptophan biosynthesis, chorismate => tryptophan_5 | organic_nitrogren | Amino Acid | Tryptophan biosynthesis, chorismate => tryptophan | 3.62E-13 | 2.802752217 |
| anthranilate synthase [EC:4.1.3.27] [RN:R00985 R00986] | Tryptophan biosynthesis, chorismate => tryptophan_4 | organic_nitrogren | Amino Acid | Tryptophan biosynthesis, chorismate => tryptophan | 5.04E-09 | 2.703277705 |
| Catlytic type: Metallo; a member is flagellinolysin (Clostridium haemolyticum) | flagellinolysin_66 | organic_nitrogren | Peptidase | flagellinolysin | 0.025108274 | 2.243777058 |
| Catlytic type: Metallo | Peptidase_63 | organic_nitrogren | Peptidase | Peptidase | 0.000692745 | 2.230267272 |
| Catlytic type: Metallo; substrates of the type Xaa-YaaZaa, with blocked amino terminus and free C-terminus ; involved in bacterial cell wall biosynthesis and metabolism | Metallopeptidases, mostly specialised carboxypeptidases and dipeptidases_62 | organic_nitrogren | Peptidase | Metallopeptidases, mostly specialised carboxypeptidases and dipeptidases | 0.000318425 | 2.073983419 |
| Catlytic type: Aspartate; HycI endopeptidase releases a 32-residue C-terminal peptide by cleavage of an -Arg-Met- bond;HybD cleaves following the equivalent -His-Met- bond in hydrogenase 2, removing 15 residues; HycI and HybD are in the processing of the precursors of bacterial hydrogenases to their active forms | Endopeptidases_61 | organic_nitrogren | Peptidase | Endopeptidases | 0.00029316 | -2.15788146 |
| amino-acid N-acetyltransferase [EC:2.3.1.1] [RN:R00259] | Ornithine biosynthesis, glutamate => ornithine_3 | organic_nitrogren | Amino Acid | Ornithine biosynthesis, glutamate => ornithine | 0.006703486 | -2.351735779 |
| ahbAB; siroheme decarboxylase [RN:R12000] | Heme biosynthesis, archaea, siroheme => heme_44 | MISC | Miscellaneous | Heme biosynthesis, archaea, siroheme => heme | 1.86E-08 | -4.336875414 |
| heterodisulfide reductase 2 [EC:1.8.7.3 1.8.98.4 1.8.98.5 1.8.98.6] [RN:R11928 R11931 R11943 R11944] | Methanogenesis, methylamine/dimethylamine/trimethylamine => methane_14 | energy | C1-methane | Methanogenesis, acetate/methanol/C02/methylamine/dimethylamine/trimethylamine => methane | 9.59E-16 | -4.016974744 |
| ech hydrogenase subunit E | hydrogenase_43 | energy | Hydrogenases | hydrogenase | 6.48E-12 | -4.281380225 |
| sulfite reductase, dissimilatory-type [EC:1.8.99.5] [RN:R00861] | Dissimilatory sulfate reduction, sulfate => H2S_68 | energy | Sulfur | Dissimilatory sulfate reduction, sulfate => H2S | 6.24E-08 | -4.392800641 |
| pyruvate carboxylase subunit A [EC:6.4.1.1] [RN:R00344] | Reductive citrate cycle (Arnon-Buchanan cycle)_10 | energy | C1 | Reductive citrate cycle (Arnon-Buchanan cycle) | 2.29E-11 | -5.278496783 |
| CBM57 Created from reading Schallus et al (2008) Mol Biol Cell. 19:3404-3414 [PMID: 18524852] and finding related domains attached to various glycosidases. | CAZYmes_Carbohydrate-Binding Modules_41 | carbon_utilization | CAZY | Carbohydrate-Binding Modules | 2.49E-14 | 5.986712111 |
| GH99 glycoprotein endo-alpha-1,2-mannosidase (EC 3.2.1.130); mannan endo-1,2-alpha-mannanase (3.2.1.-) | CAZYmes_Glycoside Hydrolases_39 | carbon_utilization | CAZY | Glycoside Hydrolases | 8.13E-13 | 5.29969753 |
| PL26 rhamnogalacturonan exolyase (EC 4.2.2.24). | CAZYmes_Polysaccharide Lyases_40 | carbon_utilization | CAZY | Polysaccharide Lyases | 2.98E-07 | 4.984252242 |
| GH50 beta-agarase (EC 3.2.1.81). | CAZYmes_Glycoside Hydrolases_38 | carbon_utilization | CAZY | Glycoside Hydrolases | 1.06E-15 | 4.960983464 |
| PL11 rhamnogalacturonan endolyase (EC 4.2.2.23); rhamnogalacturonan exolyase (EC 4.2.2.24) | CAZYmes_Polysaccharide Lyases_37 | carbon_utilization | CAZY | Polysaccharide Lyases | 9.59E-16 | 4.593658946 |
| GH144 endo-beta-1,2-glucanase (EC 3.2.1.71); beta-1,2-glucooligosaccharide sophorohydrolase (EC 3.2.1.-) | CAZYmes_Glycoside Hydrolases_35 | carbon_utilization | CAZY | Glycoside Hydrolases | 1.65E-09 | 4.14589165 |
| GH66 cycloisomaltooligosaccharide glucanotransferase (EC 2.4.1.248); dextranase (EC 3.2.1.11). | CAZYmes_Glycoside Hydrolases_36 | carbon_utilization | CAZY | Glycoside Hydrolases | 6.04E-06 | 3.950384325 |
| CE12 pectin acetylesterase (EC 3.1.1.-); rhamnogalacturonan acetylesterase (EC 3.1.1.-); acetyl xylan esterase (EC 3.1.1.72) | CAZYmes_Carbohydrate Esterases_34 | carbon_utilization | CAZY | Carbohydrate Esterases | 4.92E-12 | 3.775776847 |
| PL8 hyaluronate lyase (EC 4.2.2.1); chondroitin AC lyase (EC 4.2.2.5); xanthan lyase (EC 4.2.2.12); chondroitin ABC lyase (EC 4.2.2.20) | CAZYmes_Polysaccharide Lyases_32 | carbon_utilization | CAZY | Polysaccharide Lyases | 3.32E-09 | 3.451837953 |
| GH49 dextranase (EC 3.2.1.11); isopullulanase (EC 3.2.1.57); dextran 1,6-alpha-isomaltotriosidase (EC 3.2.1.95); sulfated arabinan endo-1,4-beta-L-arabinanase (EC 3.2.1.-) | CAZYmes_Glycoside Hydrolases_33 | carbon_utilization | CAZY | Glycoside Hydrolases | 6.49E-06 | 3.406201493 |
| PL29 hyaluronate lyase (EC 4.2.2.1); chondroitin-sulfate ABC endolyase (EC 4.2.2.20); dermatan sulfate lyase (4.2.2.-); dermatan sulfate lyase (EC 4.2.2.-) | CAZYmes_Polysaccharide Lyases_31 | carbon_utilization | CAZY | Polysaccharide Lyases | 8.17E-06 | 3.259019765 |
| GH76 alpha-1,6-mannanase (EC 3.2.1.101); alpha-glucosidase (EC 3.2.1.20) | CAZYmes_Glycoside Hydrolases_29 | carbon_utilization | CAZY | Glycoside Hydrolases | 1.57E-10 | 3.194643781 |
| CBM62 The CBM62 module of Clostridium thermocellum Cthe_2193 protein binds galactose moieties found on xyloglucan, arabinogalactan and galactomannan. | CAZYmes_Carbohydrate-Binding Modules_30 | carbon_utilization | CAZY | Carbohydrate-Binding Modules | 2.98E-07 | 3.144905924 |
| GH42 beta-galactosidase (EC 3.2.1.23); alpha-L-arabinopyranosidase (EC 3.2.1.-) | CAZYmes_Glycoside Hydrolases_28 | carbon_utilization | CAZY | Glycoside Hydrolases | 9.59E-16 | 3.092434534 |
| GH105 unsaturated rhamnogalacturonyl hydrolase (EC 3.2.1.172); d-4,5-unsaturated beta-glucuronyl hydrolase (EC 3.2.1.-); d-4,5-unsaturated alpha-galacturonidase (EC 3.2.1.-) | CAZYmes_Glycoside Hydrolases_26 | carbon_utilization | CAZY | Glycoside Hydrolases | 1.16E-09 | 2.89954158 |
| GH117 alpha-1,3-L-neoagarooligosaccharide hydrolase (EC 3.2.1.-); alpha-1,3-L-neoagarobiase / neoagarobiose hydrolase (EC 3.2.1.-) | CAZYmes_Glycoside Hydrolases_27 | carbon_utilization | CAZY | Glycoside Hydrolases | 1.04E-08 | 2.88185501 |
| GH37 alpha,alpha-trehalase (EC 3.2.1.28). | CAZYmes_Glycoside Hydrolases_25 | carbon_utilization | CAZY | Glycoside Hydrolases | 1.54E-05 | 2.710593562 |
| GH115 xylan alpha-1,2-glucuronidase (3.2.1.131); alpha-(4-O-methyl)-glucuronidase (3.2.1.-) | CAZYmes_Glycoside Hydrolases_24 | carbon_utilization | CAZY | Glycoside Hydrolases | 5.41E-08 | 2.642120203 |
| CBM4 Modules of approx. 150 residues found in bacterial enzymes. Binding of these modules has been demonstrated with xylan, beta-1,3-glucan, beta-1,3-1,4-glucan, beta-1,6-glucan and amorphous cellulose but not with crystalline cellulose. | CAZYmes_Carbohydrate-Binding Modules_23 | carbon_utilization | CAZY | Carbohydrate-Binding Modules | 3.24E-06 | 2.403605583 |
| GH28 polygalacturonase (EC 3.2.1.15); alpha-L-rhamnosidase (EC 3.2.1.40); exo-polygalacturonase (EC 3.2.1.67); exo-polygalacturonosidase (EC 3.2.1.82); rhamnogalacturonase (EC 3.2.1.171); rhamnogalacturonan alpha-1,2-galacturonohydrolase (EC 3.2.1.173); endo-xylogalacturonan hydrolase (EC 3.2.1.-) | CAZYmes_Glycoside Hydrolases_22 | carbon_utilization | CAZY | Glycoside Hydrolases | 6.02E-07 | 2.367984994 |
| GH43 beta-xylosidase (EC 3.2.1.37); alpha-L-arabinofuranosidase (EC 3.2.1.55); xylanase (EC 3.2.1.8); alpha-1,2-L-arabinofuranosidase (EC 3.2.1.-); exo-alpha-1,5-L-arabinofuranosidase (EC 3.2.1.-); [inverting] exo-alpha-1,5-L-arabinanase (EC 3.2.1.-); beta-1,3-xylosidase (EC 3.2.1.-); [inverting] exo-alpha-1,5-L-arabinanase (EC 3.2.1.-); [inverting] endo-alpha-1,5-L-arabinanase (EC 3.2.1.99); exo-beta-1,3-galactanase (EC 3.2.1.145); beta-D-galactofuranosidase (EC 3.2.1.146) | CAZYmes_Glycoside Hydrolases_19 | carbon_utilization | CAZY | Glycoside Hydrolases | 1.26E-09 | 2.343978898 |
| CBM6 Modules of approx. 120 residues. The cellulose-binding function has been demonstrated in one case on amorphous cellulose and beta-1,4-xylan. Some of these modules also bind beta-1,3-glucan, beta-1,3-1,4-glucan, and beta-1,4-glucan. | CAZYmes_Carbohydrate-Binding Modules_20 | carbon_utilization | CAZY | Carbohydrate-Binding Modules | 1.51E-05 | 2.330679483 |
| GH154 beta-glucuronidase (3.2.1.31) | CAZYmes_Glycoside Hydrolases_21 | carbon_utilization | CAZY | Glycoside Hydrolases | 0.000332397 | 2.262150583 |
| GH10 endo-1,4-beta-xylanase (EC 3.2.1.8); endo-1,3-beta-xylanase (EC 3.2.1.32); tomatinase (EC 3.2.1.-); xylan endotransglycosylase (EC 2.4.2.-); endo-beta-1,4-glucanase (EC 3.2.1.4) | CAZYmes_Glycoside Hydrolases_17 | carbon_utilization | CAZY | Glycoside Hydrolases | 3.15E-07 | 2.253506629 |
| GH67 alpha-glucuronidase (EC 3.2.1.139); xylan alpha-1,2-glucuronidase (EC 3.2.1.131) | CAZYmes_Glycoside Hydrolases_18 | carbon_utilization | CAZY | Glycoside Hydrolases | 0.000334531 | 2.214187127 |
| fructose-1,6-bisphosphatase [EC:3.1.3.11] [RN:R04780] | Gluconeogenesis, oxaloacetate => fructose-6P_42 | carbon_utilization | Central carbon | Gluconeogenesis, oxaloacetate => fructose-6P | 3.20E-09 | -4.227019461 |

The information of the gene including a KEGG number, description, DRAM category and subcategory, and KEGG module is shown. Additionally the log2 fold change (FC) in the mean abundance of high fibre-fed mice compared to low fibre-fed mice, and FDR adjusted p-values are presented per each DA bacterial-encoded gene. N=4 mice/group.

**Supplementary figures**

**
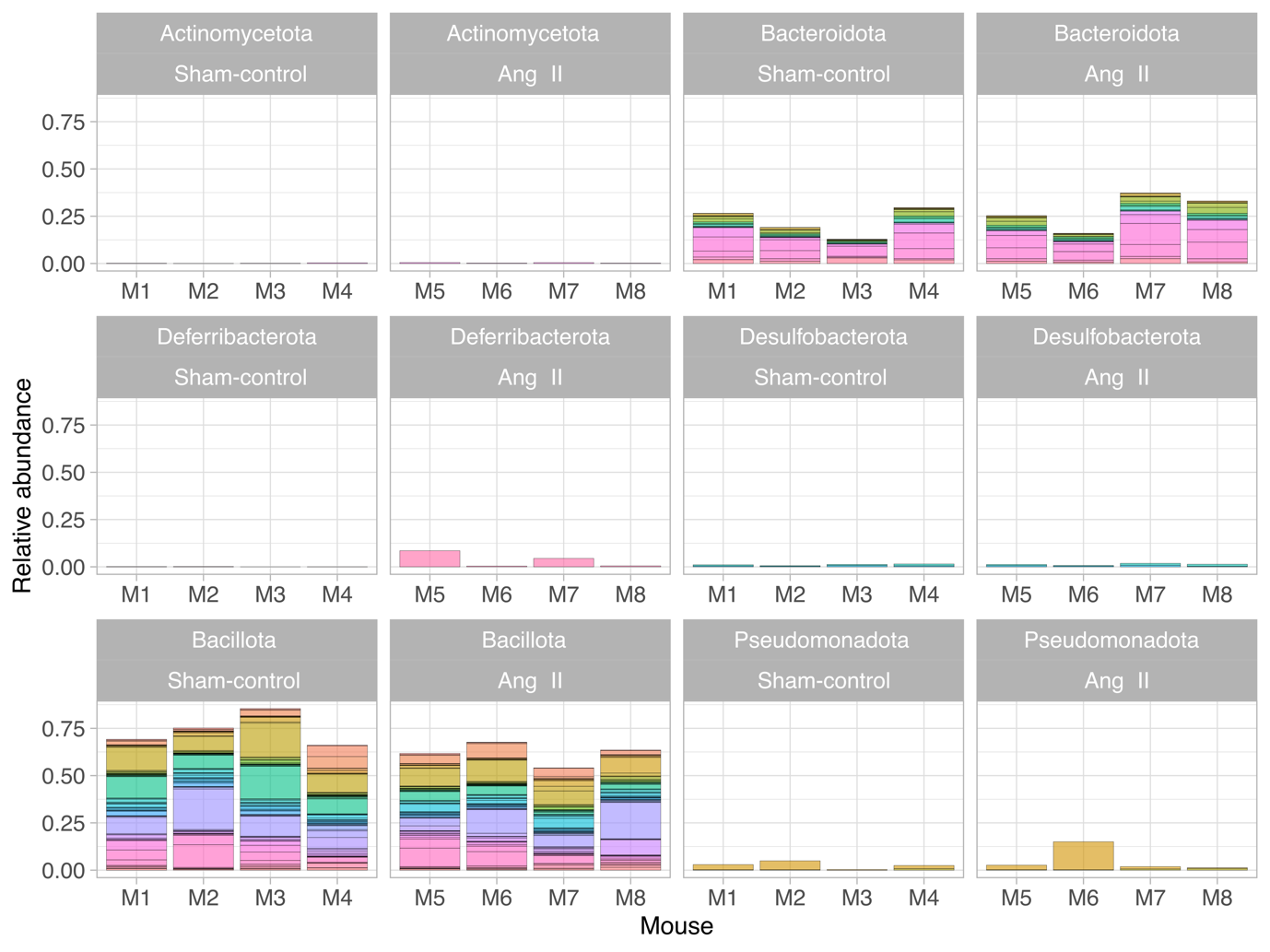
**

**Supplementary Figure 1. Taxonomic composition of gut bacterial populations in Ang II-challenged and Sham-control mice.** The relative abundance of each bacterial SRG is represented by each black-delimited sub-bar; the colour of each SRG (sub-bar) was assigned randomly. For readability, the stacked bar chat is subdivided at the phylum level.


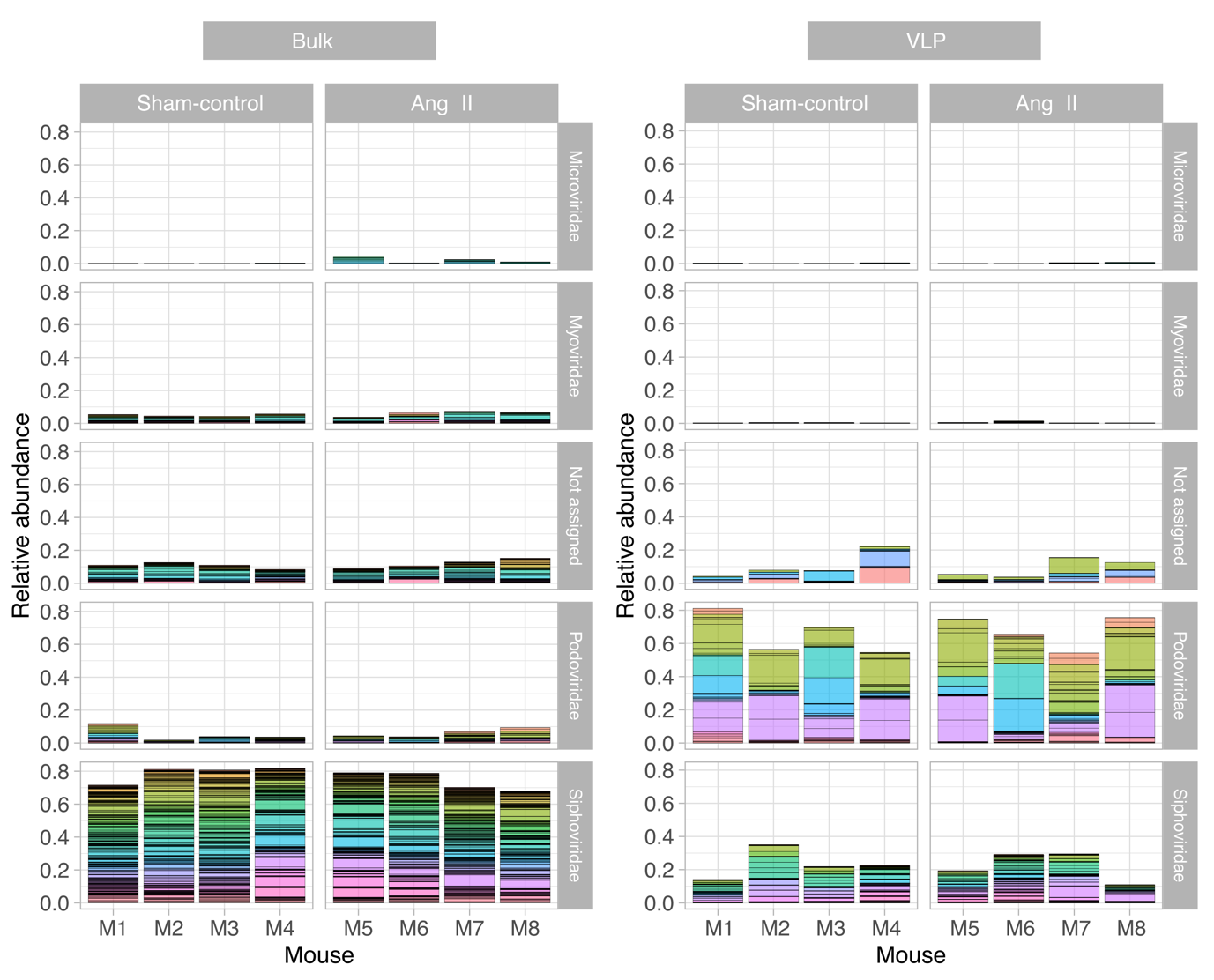


**Supplementary Figure 2. Taxonomic composition of gut viral populations in Ang II-challenged and Sham-control mice.** The relative abundance of each viral SRG is represented by each black delimited sub-bar, the colour of each SRG (sub-bar) was assigned randomly. For readability the stacked bar chat is sub-divided at Family-level.


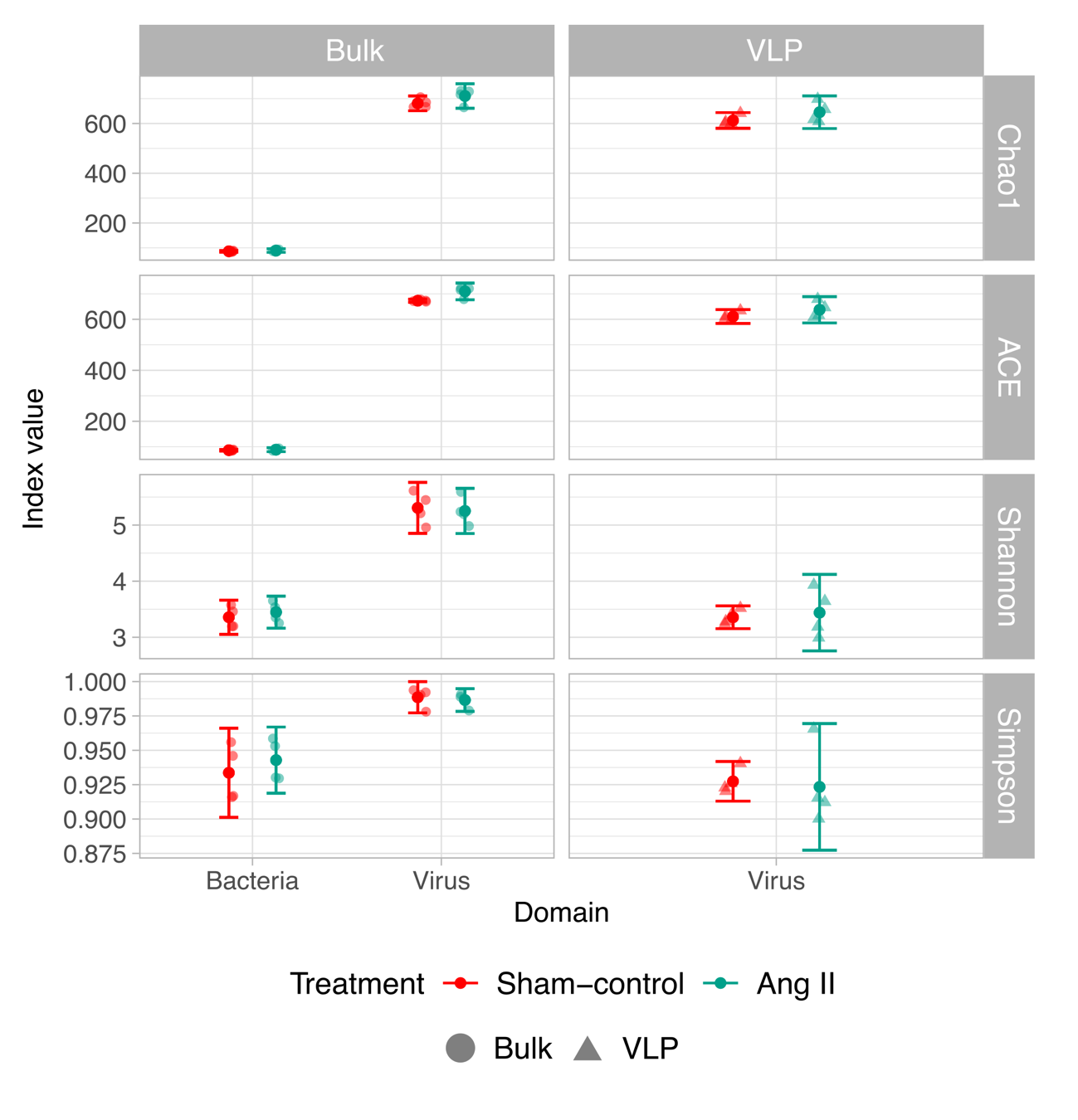


**Supplementary Figure 3. Alpha-diversity indices did not change between normotensive and Ang II-driven hypertensive mice.** After 4-weeks of implantation of a minipump either with saline (Sham-control) or Ang II, ACE, Chao1, Simpson, and Shannon alpha-diversity index of gut bacterial and viral populations did not change. N=4 mice/group. Two-pair group t-test. The dark points represent the mean, the translucid dots represent the measure of each mouse, and the whiskers represent the 95% CI.

**
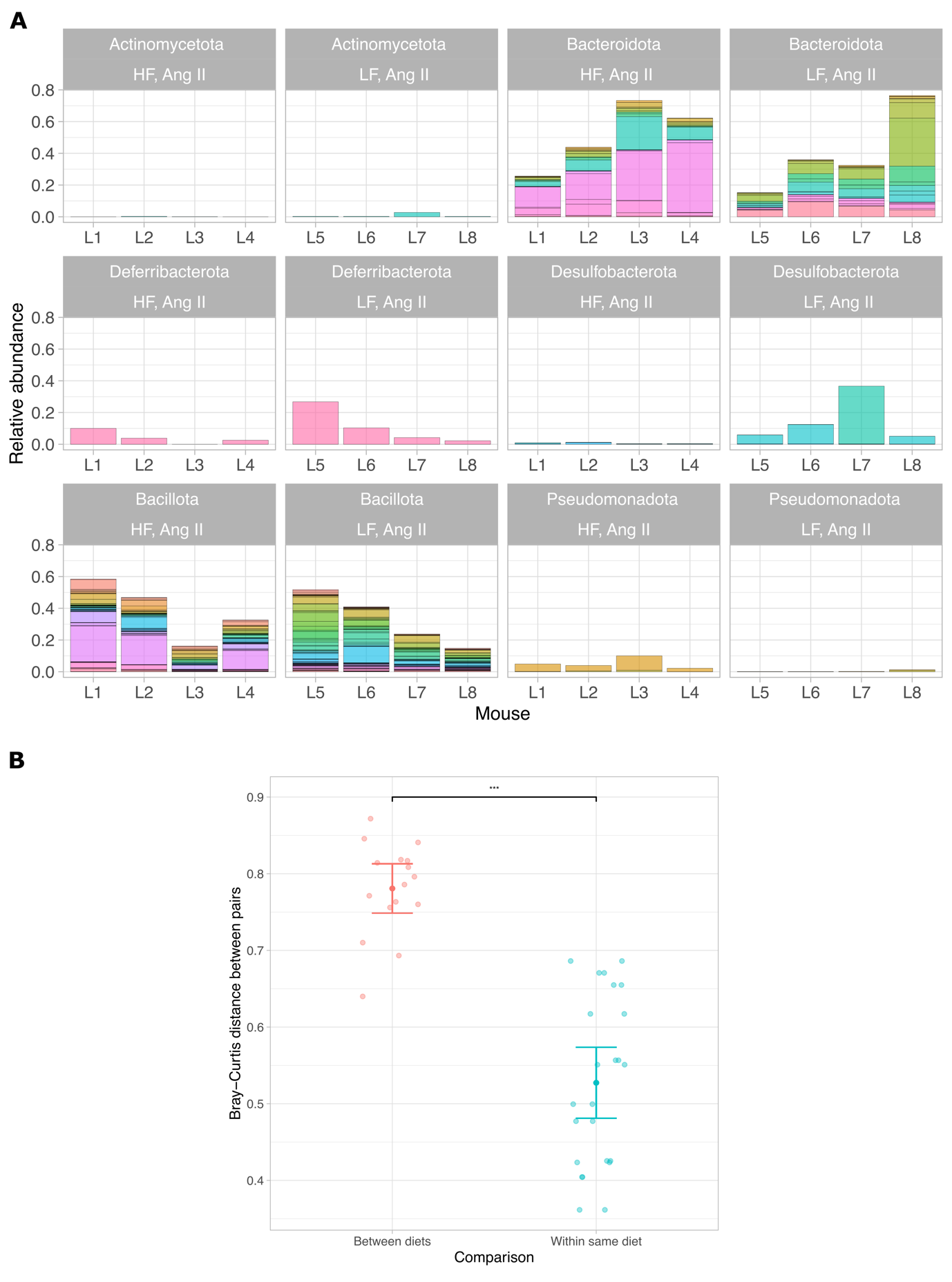
**

**Supplementary Figure 4. Taxonomic composition of gut bacterial populations in high-fibre-fed and low-fibre-fed Ang II challenged mice. A**, the relative abundance of each bacterial SRG is represented by each black delimited sub-bar. The colour of each SRG (sub-bar) was assigned randomly, so they are not comparable with colours in Supplementary Figure 1. For readability the stacked bar chat is sub-divided at Phylum-level. The colours of this figure. **B.** Bray-Curtis distances of bacterial communities between pairs of mice from the same diet (within same diet) or between diets. *P<0.05; **P<0.01; ***P<0.001; NS P≥0.05. Pairwise Wilcoxon test with FDR corrections for multiple comparisons**.** The dark points represent the mean, the whiskers represent the 95% CI, and the translucid dots represent the distance from each mice-pair comparison.


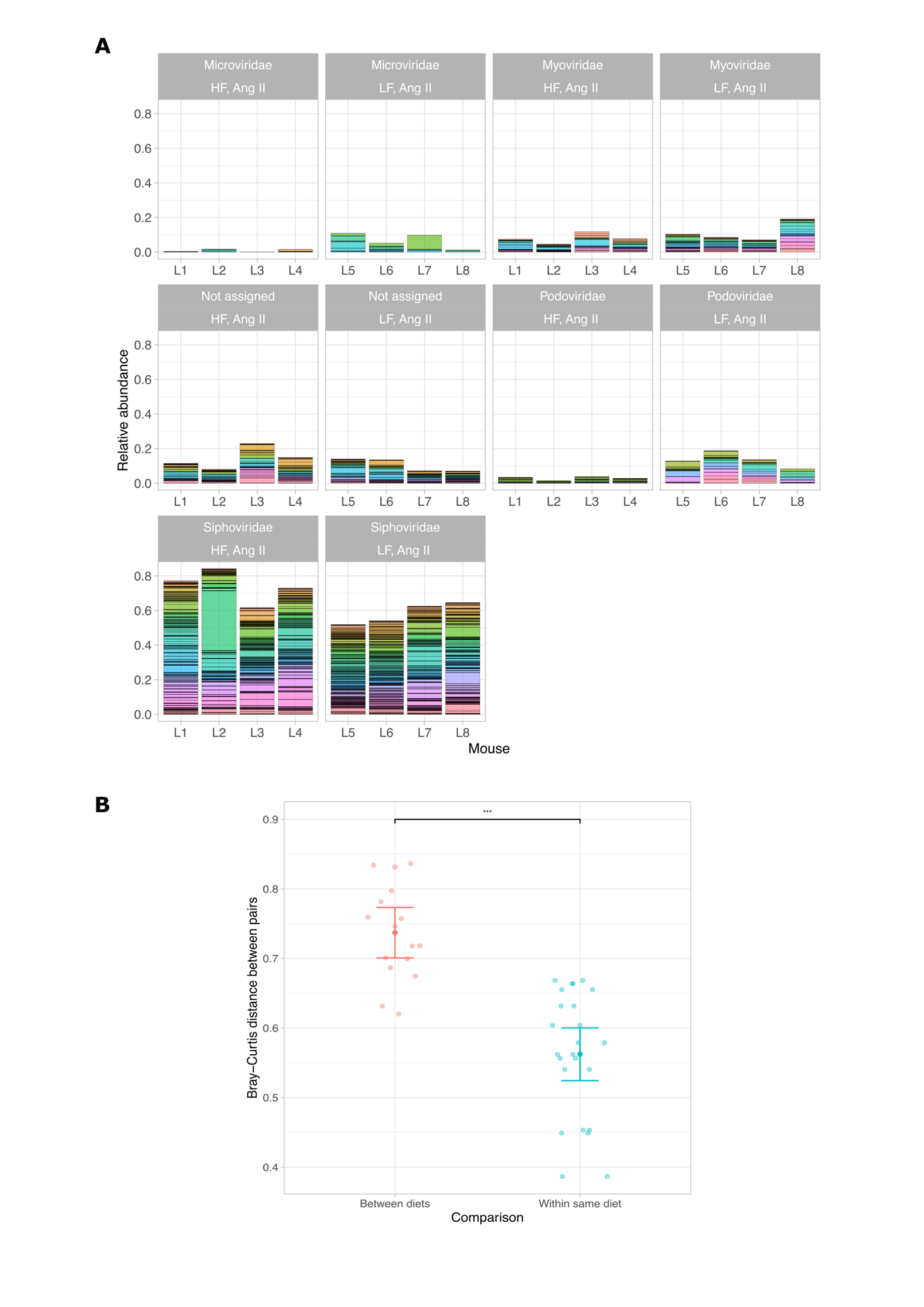


**Supplementary Figure 5. Taxonomic composition of gut viral populations in high-fibre-fed and low-fibre-fed Ang II challenged mice.**  **A,** the relative abundance of each viral SRG is represented by each black delimited sub-bar. The colour of each SRG (sub-bar) was assigned randomly, so they are not comparable with colours in Supplementary Figure 2. For readability the stacked bar chat is sub-divided at Family-level. **B,** Bray-Curtis distances of viral communities between pairs of mice from the same diet (within same diet) or between diets. *P<0.05; **P<0.01; ***P<0.001; NS P≥0.05. Pairwise Wilcoxon test with FDR corrections for multiple comparisons**.** The dark points represent the mean, the whiskers represent the 95% CI, and the translucid dots represent the distance from each mice-pair comparison.

**
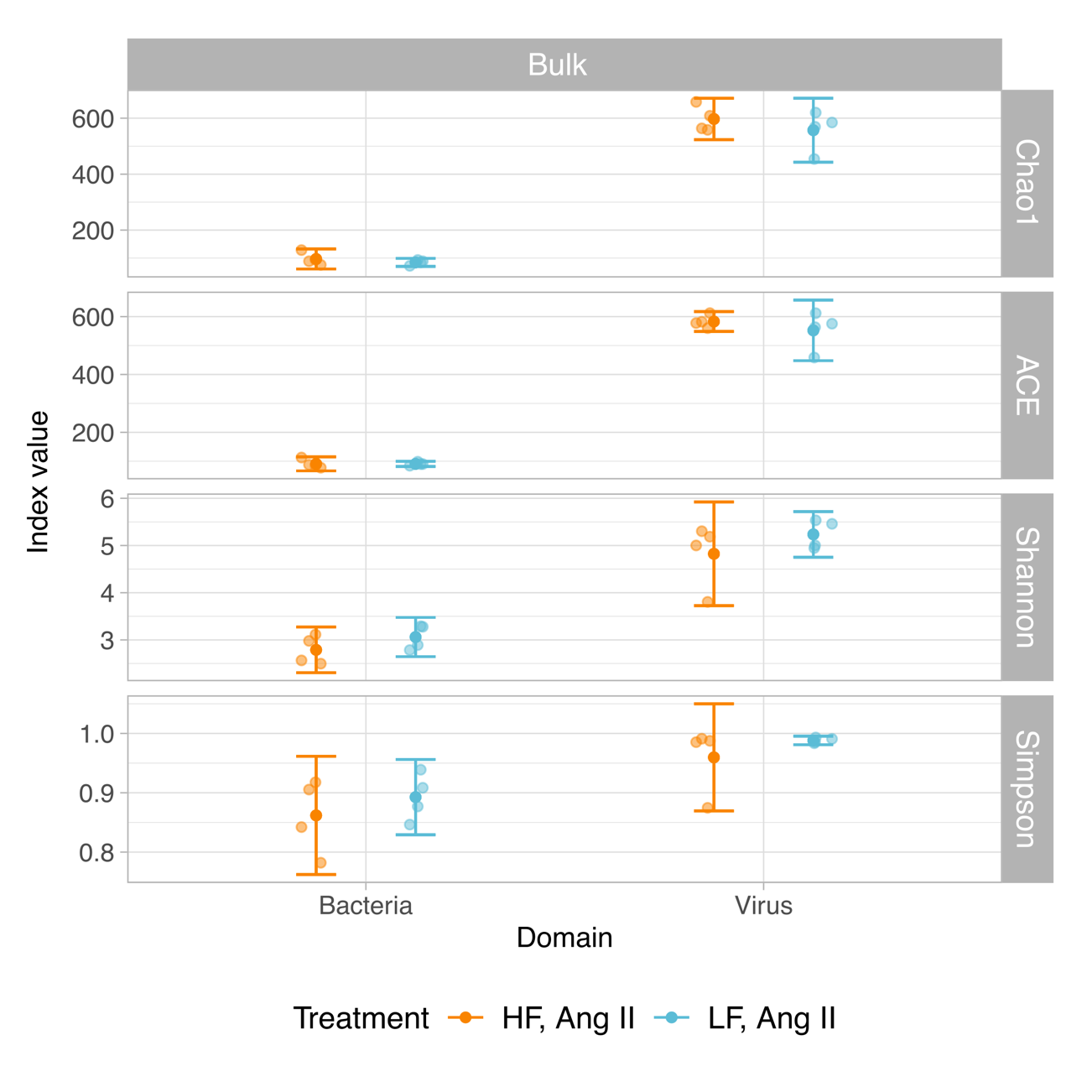
**

**Supplementary Figure 6. Alpha-diversity indices did not change between high-fibre and low-fibre fed Ang II-mice.** After 4-weeks of implantation of a minipump with Ang II, ACE, Chao1, Simpson, and Shannon alpha-diversity index of gut bacterial and viral populations did not change between fibre-intake groups. N=4 mice/group. Two-pair group t-test. The dark points represent the mean, the translucid dots represent the measure of each mouse, and the whiskers represent the 95% CI.

**
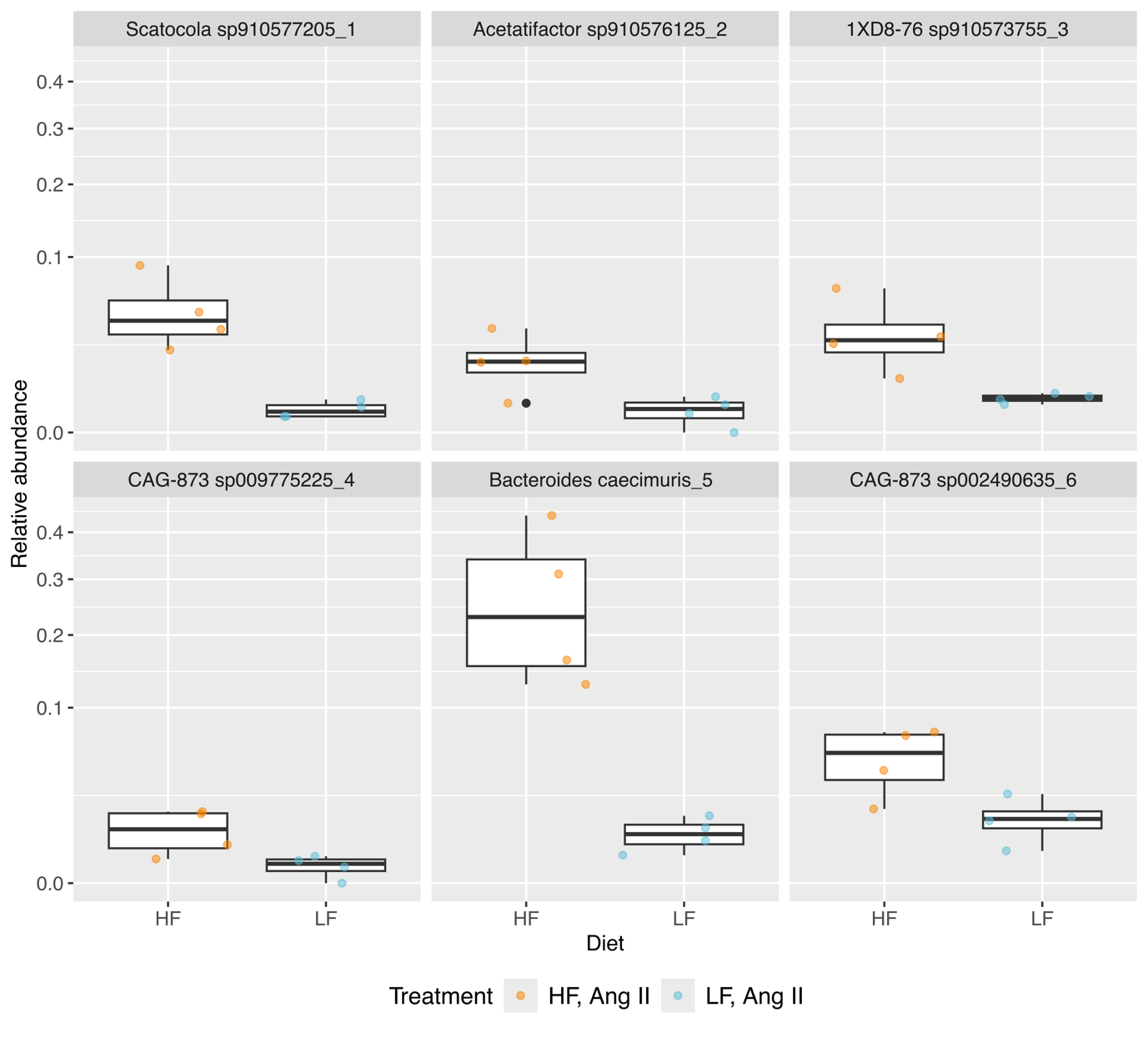
**

**Supplementary Figure 7. Relative abundance of differently abundant (DA) bacterial SRGs**. Each plot corresponds to a DA bacterial SRG. The translucid dots represent the value per mouse.

**
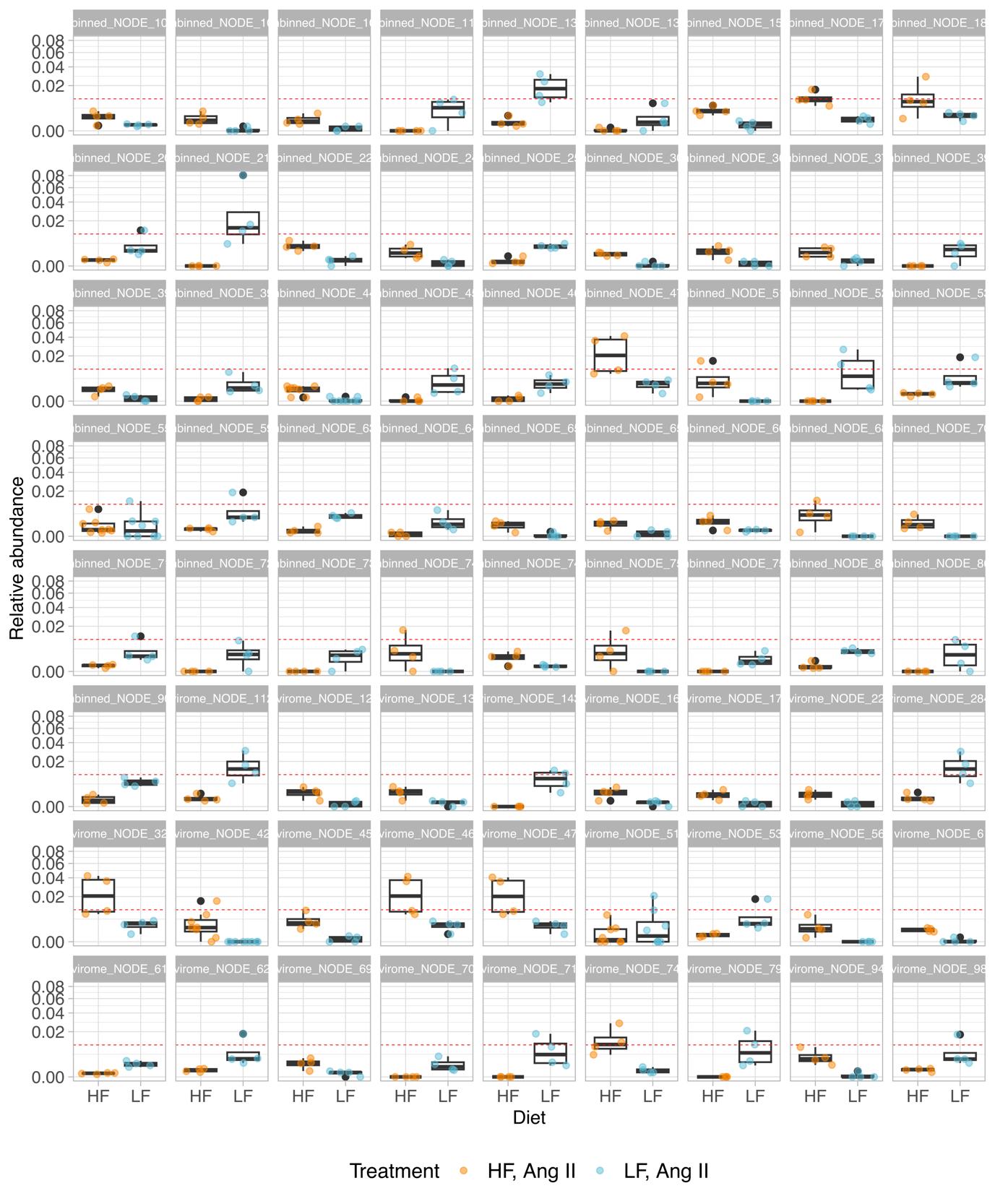
**

**Supplementary Figure 8. Relative abundance of differently abundant (DA) viral SRGs**. Each plot corresponds to a DA bacterial SRG. The translucid dots represent the value per mouse.

**
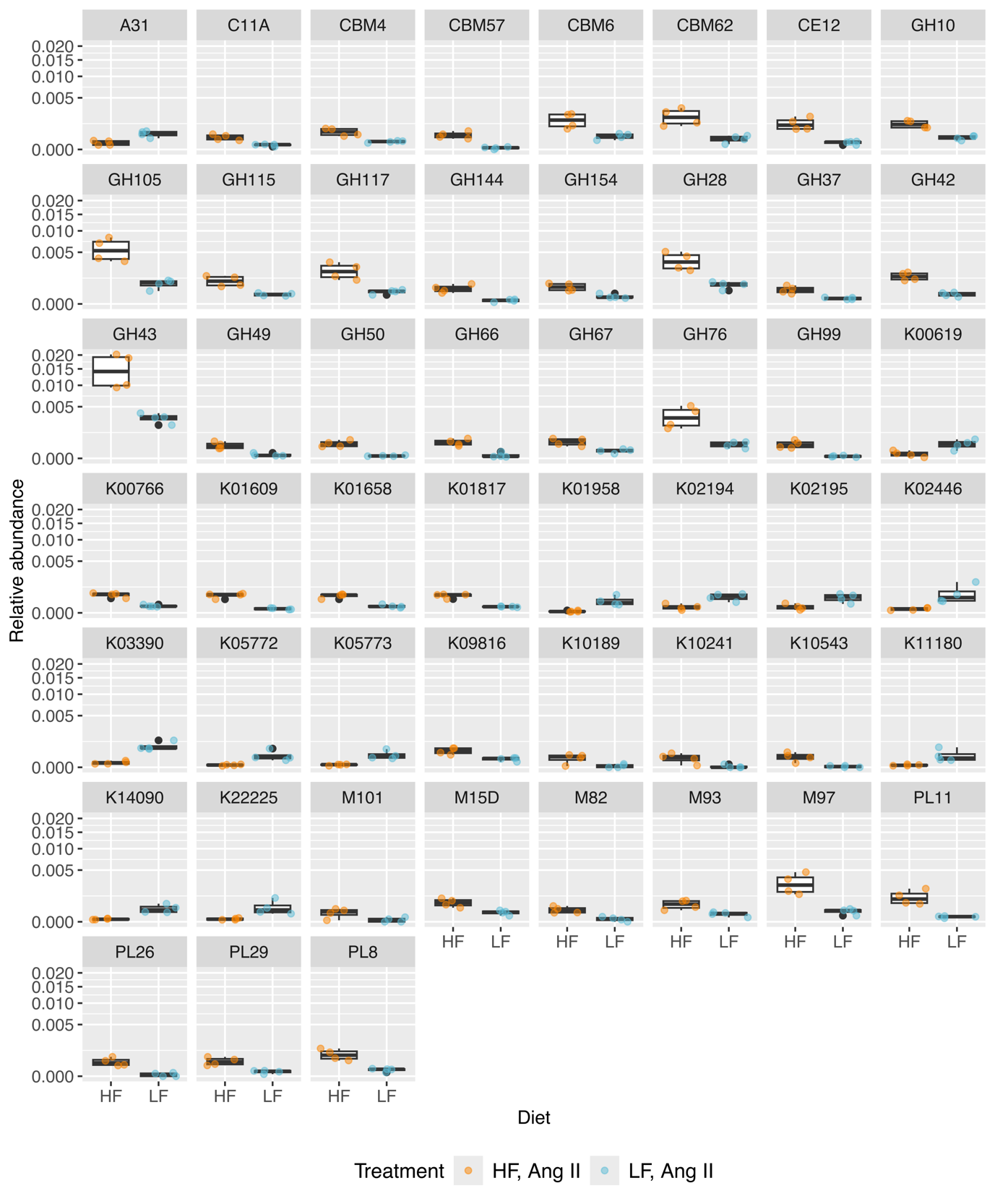
**

**Supplementary Figure 9. Relative abundance of differently abundant (DA) bacterial-encoded genes**. Each plot corresponds to a DA bacterial SRG. The translucid dots represent the value per mouse.

**
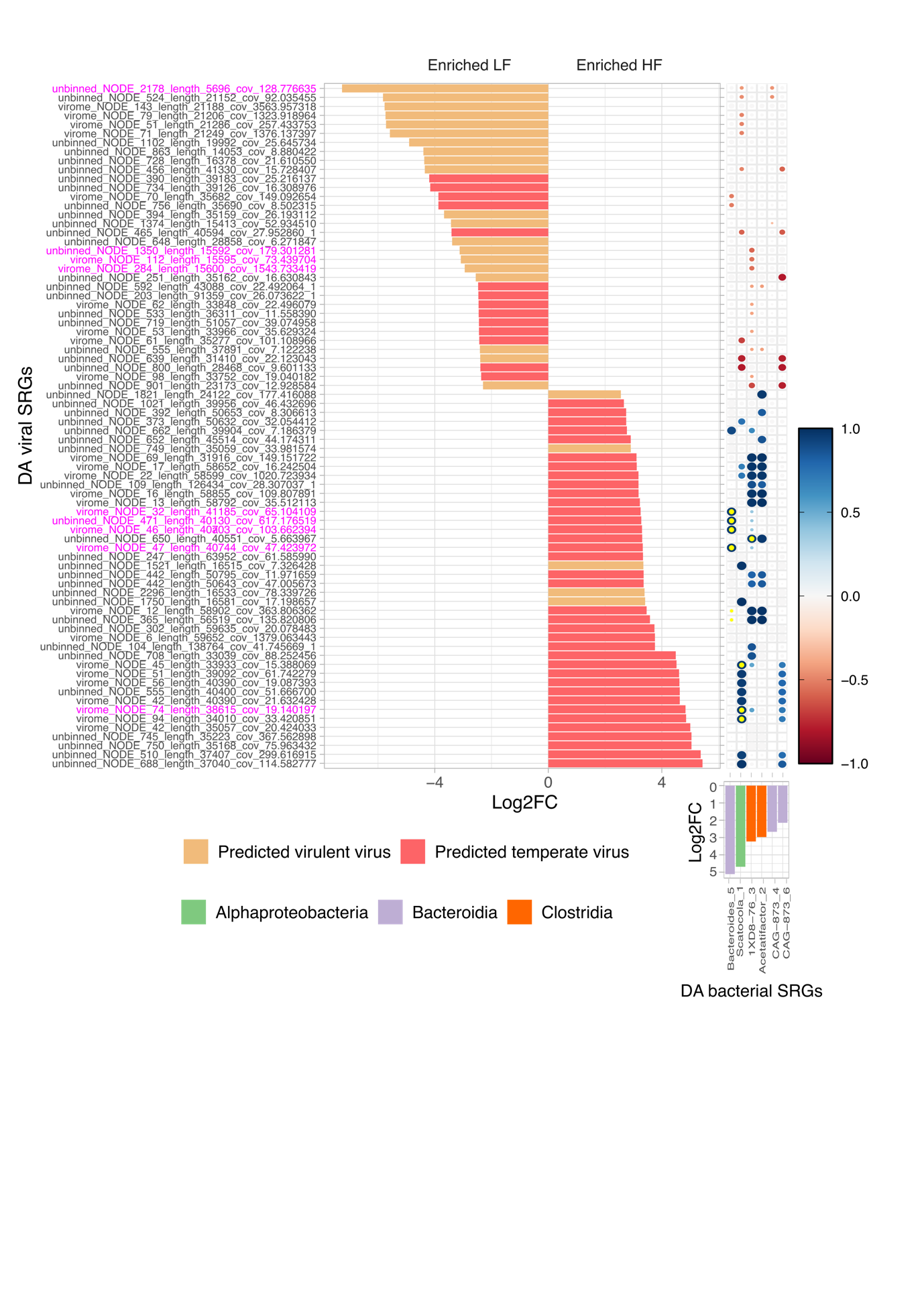
**

**Supplementary Figure 10. Differently abundant (DA) viral SRGs between high fibre and low fibre fed mice.** DA viral SRGs (left panel), DA bacterial SRGs (bottom right panel), and their correlation (top right panel) are present. In the left panel the log2 fold change in mean abundance of viral SRGs that are differently abundant in high fibre-fed mice compared to low fibre-fed mice are shown. Positive coefficient indicates the enrichment in HF-fed-mice gut bacterial community. Viral SRGs are coloured by their predicted lifestyle, and the ones written in fuchsia had a relative abundance higher to 0.01 in at least two samples of the same treatment. Similarly, the log2 fold change in mean abundance of bacterial SRGs that are differently abundant in the bacterial community of high fibre-fed mice compared to low fibre-fed mice is presented in the bottom right panel and coloured based on bacterial phylogenetic class. Spearman correlation coefficients of only significance correlations between DA viral and DA bacterial SRGs is presented in the top right panel. Given a bacteria-virus pair, a central yellow point in the correlation plot represents when at least 50% of that viral genome was detected in that bacterial genome. Consensus differential features with | log2FC | > 2 from DeSeq, MaAsLin, and metagenomeSeq outputs using dar (**A**).^48^ Spearman correlation test with FDR corrections for multiple testing **(A)**, pairwise t-test with FDR corrections for multiple testing.
